# Study on the proportion of paste filling materials based on fluorogypsum

**DOI:** 10.1371/journal.pone.0286872

**Published:** 2023-06-08

**Authors:** Wang Chun, Li Xin-ru, Cheng Lu-ping, Xiong Zu-qiang, Zhan Shuai-fei

**Affiliations:** 1 Sinosteel MaAnShan General Institute of Mining Research Co., LTD, Maanshan Anhui, China; 2 School of Energy Science and Engineering, Henan Polytechnic University, Jiaozuo Henan, China; 3 Collaborative Innovation Center of Coal Work Safety and Clean High Efficiency Utilization, Jiaozuo Henan, China; Hacettepe Universitesi, TURKEY

## Abstract

A new type of paste filling material was created using fluorogypsum, a byproduct of hydrofluoric acid, as the raw material to address the issue of the filling material’s high cost. The effects of five factors, including gangue, fly ash, fluorogypsum, lime content, and mass concentration on the physical and mechanical properties of filling material were also examined. In addition to analyzing slump and extension changes, the filler’s mineral composition and microstructure were examined using SEM and XRD examinations. The results show that the best ratio of the developed filling material was 1000g coal gangue, 300g fly ash, 300g fluorogypsum, and 50g lime and mass concentration is 78%, and its compressive strength can reach 4–5MPa at 28 days. Raw materials such as gangue and fly ash will have a certain influence on the mechanical properties of the filling material. The hydration products of the developed filling material prepared by XRD and SEM were ettringite, calcium sulfate dihydrate, and calcium silicate hydrate gel. The new fluorogypsum-based paste filling material can be used to consolidate loose rock strata and fill goaf. It solves the problem of disposal of industrial waste fluoropgypsum and also solves the problem of coal mine gangue stacking, which has a far-reaching influence on ecological environment management.

## 1 Introduction

Filling mining is an important part of green mining. It not only solves the problems of mine waste accumulation and disposal of polluting waste from surrounding factories but also protects the natural ecological environment and improves the coal resource recovery rate [1–4]. All the advantages mentioned above indicated that filling mining has become the main means for ‘three unders’ in coal mining, goaf treatment, rock movement control, and surface subsidence mitigation in China at present [5–8]. To date, more than 200 green mines have been built and approximately 1.479 billion tons of mine wastes have been processed. As the core of filling mining, filling material is an important factor to determine the cost and effect of backfill, under the premise of ensuring the filling effect, reducing the filling cost is an important means to improve the cost performance of filling mining. Especially the coal gangue produced in coal mining cannot meet the needs of goaf filling, which has become the bottleneck problem that needs to be solved urgently. Based on this problem, it is urgent to develop new paste fillers with sufficient raw materials and low cost, which also has certain scientific significance and application prospect.

Based on the collaborative solution of mine waste rock and industrial waste, the exploration of new paste materials suitable for coal mine goaf filling and the study of new paste fillers based on fluorine gypsum industrial waste can provide new ideas for solving the serious shortage of coal mine filling materials. The industrial waste fluorogypsum (FG), which has adverse effects on people and the environment, is composed of calcium sulfate (CaSO_4_), calcium fluoride (CaF_2_), and a small amount of sulfuric acid (H₂SO₄) and hydrofluoric acid (HF) [9]. However, for every 1 ton of HF produced, 3.6 tons of FG is generated [10]. The disposal of FG brings great pressure to the relevant enterprises. Due to the low solubility of FG, its coagulation rate is slow and the early hydration and hardening degree are low. These adverse factors will greatly limit the development and application of FG [11, 12]. Therefore, scholars at home and abroad have conducted in-depth research and achieved some results. Sandra L. Rodríguez R et al. [13] utilized FG for the chemical surface treatment of rubber tire powder used as aggregate for cementing mixture, which improved its interaction with the cement matrix. Xuquan Huang et al. [14–17] developed an FG-based binder using FG as a cementing material and improved the strength of the material at different ages. Yasser Bigdeli et al. [18–21] developed a new FG-Fly ash-Portland cement blend. This FG-based mixture is a promising low-cost construction material for outdoor and underwater construction. Zhong Wu et al. [22] applied FG in subgrade construction and solved its moisture-proof problem. Zhang Kang et al. [23, 24] studied the chemical composition, mineralogical properties, and thermal dynamic characteristics of water-saturated FG, and improved its early mechanical strength through modification treatment. To sum up, at present, the main treatment methods of fluorine gypsum are to stimulate the activity of fluorine gypsum, to be used as retarding agent of cement, building materials, to produce potassium sulfate and aluminum sulfate, etc., but the amount is small, which cannot match the production of fluorine gypsum waste. At the same time, there are few researches on the application of fluorine gypsum in mine filling. It has objective environmental, economic and social benefits to use fluorine gypsum as raw materials to replace desulfurization gypsum and cement in paste fillers.

According to the problems of a large number of goaf left by coal mining, serious shortage of filling materials and urgent solution and utilization of industrial waste fluorine gypsum, a new type of fluorine gypsum based paste filling material is developed by combining the two, which is one of the effective ways to solve the above problems. On the one hand, the mine has a great demand for filling materials, and the price of fluorine gypsum is low, which has economic value as filling raw materials. On the other hand, fluorine gypsum is a kind of toxic and harmful pollution waste, which can be transformed into non-toxic and harmless substances through modification. It can not only solve the problem of ecological environment destruction caused by stacking, but also has high social benefits. Therefore, the study of new filling materials with fluorine gypsum as the main component can well expand the application range of fluorine gypsum, solve the problem of a large number of fluorine gypsum nowhere to be stacked, realize the win-win situation of fluorine gypsum waste utilization and efficient filling in coal mines, and achieve the purpose of protecting the natural ecological environment and reducing the cost of filling materials.

## 2 Raw materials and test methods

The basic idea of developing new paste filling material based on the industrial by-product FG was to use coal gangue, fly ash (PFA), and FG as the main materials, with a certain amount of additives, and to prepare paste filling material by the trial mixing method. Then, the uniaxial compressive strength of the prepared square specimens was used as a parameter to determine the optimum paste filling material ratio. Finally, the fluidity and compressive strength of the developed paste filling material were measured, and the microscopic composition of the material was analyzed in combination with XRD and SEM tests to reveal the formation mechanism of the developed new paste filling material.

### 2.1 Test materials

To ensure high-purity raw materials, all raw materials selected for testing were chosen directly from the supplier’s factory. Gangue, PFA, FG as the main material, lime as the auxiliary material, early strength agent, and water reducing agent as additives together formed the raw material of the new FG-based paste filling material. Meanwhile, FG was replaced by sulfoaluminate cement and desulfurization gypsum (FGD) as the main substitutes and the performance of FG-based paste filling material was compared and analyzed. The main chemical composition, content, and supplier of each raw material are shown in Table 1.

**Table 1:** The main chemical composition, content (wt. %), and supplier of each raw material.

Chemical compositions	SiO_2_	Al_2_O_3_	Fe_2_O_3_	CaO	MgO	SO_3_	LOI	MgO	Total other components (Individual content <0.5%)	Suppliers
PFA	52.28	27.32	3.65	1.21	3.72	2.15	1.29	/	8.38	Jiaozuo Power Plant
FG	4.85	2.64	0.21	35.84	1.05	53.19	1.27	/	0.95	Dovetail Corporation
Sulfoaluminate Cement	21.62	4.65	3.12	59.36	2.12	2.58	1.08	/	5.47	Dengfeng Power Group
FGD	3.55	1.07	0.72	32.36	0.42	42.58	2.64	/	16.66	Jinan Steel Group
Lime	/	/	/	80.35	/	/	/	5.32	14.33	Yusheng Calcium Industry

Coal gangue as the main material was provided by SiHe Mine in Shanxi Province. It had no chemical reaction with other raw materials, but its particle size had a certain impact on the fluidity and compressive strength of the paste filling material. EP-1/100x125 small jaw crusher was adopted and screened to obtain the particle size distribution as shown in Table 2.

**Table 2:** Particle size distribution of coal gangue.

Particle size /mm	<0.15	0.15~0.3	0.3~0.6	0.6~1.18	1.18~2.36	2.36~4.75	4.75~9.5	>9.5
Ratio/%	3.16	8.54	17.71	18.26	21.15	23.57	4.58	3.03

### 2.2 Test methods

#### 2.2.1 Test equipment and process

The research of new paste filling material based on industrial by-product FG mainly involved four tests of material trial formulation, compressive strength test, SEM, and XRD. In the experimental preparation of FG-based new paste filling material, tap water was poured into the mixture of gangue, PFA, FG, lime, and additives under the condition of room temperature of 20°C in accordance with the proposed ratio. After stirring well, it was poured into the specimen mold of 70.7mm×70.7mm×70.7mm to prepare the specimens of FG-based paste filling material. The specimens were demoulded after 24h of resting, and the demoulded specimens were wrapped with cling film and placed in a presetting maintenance box with the temperature set at 20°C and humidity at 95%. The curing ages of the specimens were set to 1 day, 7 days, and 28 days. The specimens meeting the test requirements were selected and placed on the test platform of the YAW-300 microcomputer-controlled electro-hydraulic servo pressure testing machine for a uniaxial compressive strength test. The loading method was force loading, and the loading speed was 0.5kN/s. The average of three valid data for each group of ratios was taken as the final result to reduce the testing error.

**Fig 1:**
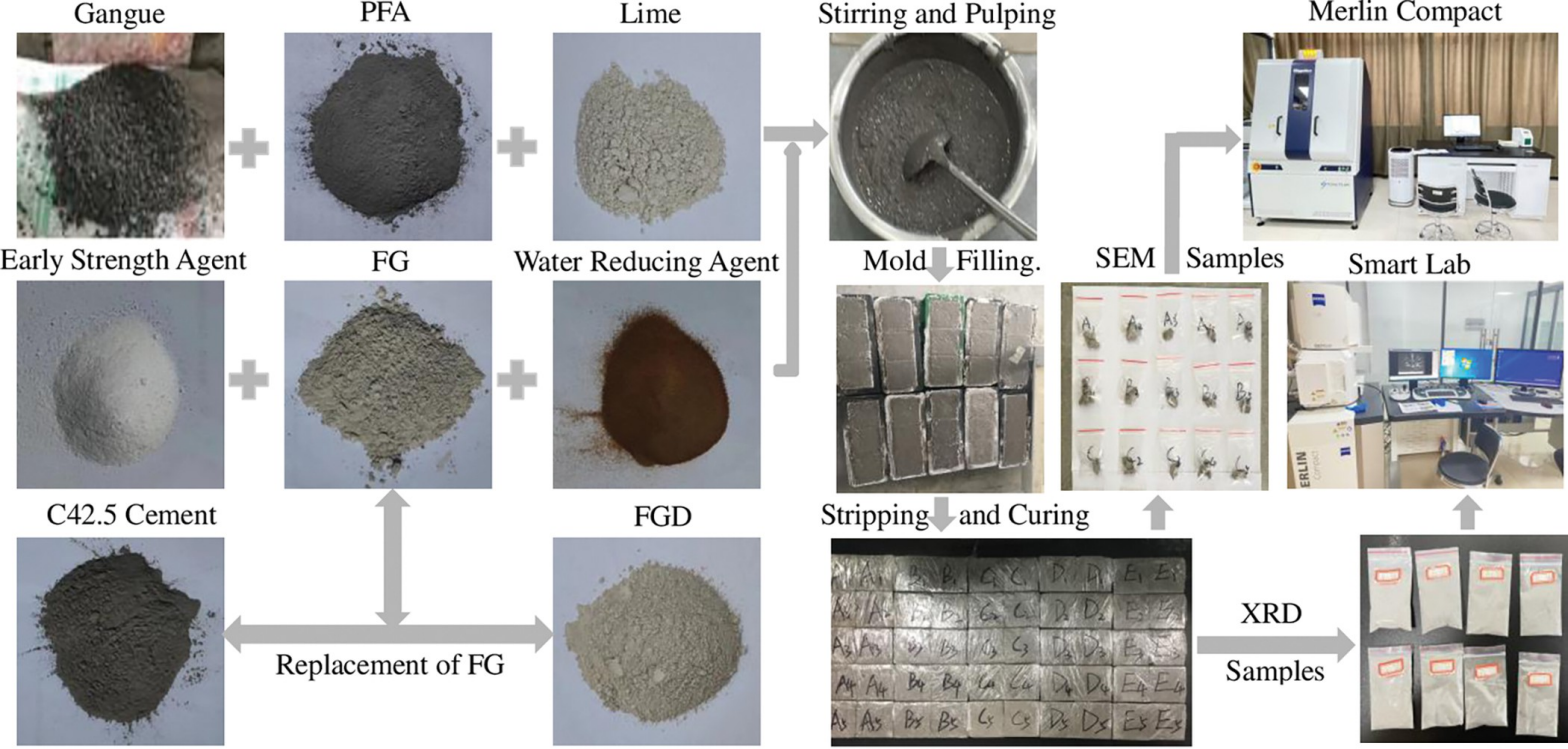
Research process of new paste filling material based on industrial by-product FG.

To analyze the final microscopic composition of the developed FG-based paste filling material, the crushed samples with a diameter of less than 5cm and height of less than 1cm were immersed in anhydrous ethanol for 24h to terminate hydration. The samples were then dried in a vacuum oven at 40°C for 2 days, and then SEM microstructure analysis tests were carried out using a German Merlin Compact scanning electron microscope with a voltage setting of 10 kV. Simultaneously, some of the dried crushed samples were placed in agate vessels and ground into a uniform powder. Then, the samples were tested by XRD using a Japanese Smart Lab instrument with a test angle of 5°-70°, a test speed of 10°/min, and a voltage and current of 40 kV and 150 mA, respectively. The specific research procedure is shown in Fig 1.

#### 2.2.2 Test scheme

In the development of new paste filling material based on industrial by-product FG, the optimal ratio range of the material was initially determined by the trial formulation method, and then the influence of each raw material on the mechanical properties of the paste filling material was studied by the single factor analysis method. Five 70.7 mm × 70.7 mm × 70.7 mm cubic specimens were prepared for each group of tests, and at least three sets of valid data were selected and averaged as the test results for analysis. The specific test scheme is shown in Table 3.

**Table 3:** Test scheme based on the trial formulation of new filling material based on industrial by-product FG and the influence of each raw material on its mechanical properties.

Test scheme for the proportioning of new paste filling material								Test scheme for each raw material affecting the performance of paste filling material					
Number	Gangue(g)	PFA(g)	Cement(g)	FG(g)	FGD(g)	Lime(g)	Mass concentration(%)	Number	Gangue(g)	PFA(g)	FG(g)	Lime(g)	Mass concentration(%)
1	1200	200	200	0	0	40	78	A1	800	300	300	50	78
2	1200	200	0	200	0	40	78	A2	900				
3	1200	200	0	0	200	40	78	A3	1000				
4	1000	300	300	0	0	100	78	A4	1100				
5	1000	300	0	300	0	100	78	A5	1200				
6	1000	300	0	0	300	100	78	B1	1000	100	300	50	78
7	1000	300	0	300	0	0	78	B2		200			
8	1000	300	0	300	0	20	78	B3		300			
9	1000	300	0	300	0	40	78	B4		400			
10	1000	300	0	300	0	60	78	B5		500			
11	1000	300	0	300	0	80	78	C1	1000	300	100	50	78
12	1000	300	0	300	0	100	78	C2			200		
13	1000	300	0	300	0	100	77	C3			300		
14	1000	300	0	300	0	100	78	C4			400		
15	1000	300	0	300	0	100	79	C5			500		
16	1000	300	0	300	0	100	80	D1	1000	300	300	10	78
17	1000	300	0	300	0	100	81	D2				30	
18	1000	300	0	300	0	50	78	D3				50	
19	1000	300	25	300	0	50	78	D4				70	
20	1000	300	50	300	0	50	78	D5				90	
21	1000	300	75	300	0	50	78	E1	1000	300	300	50	76
22	1000	300	100	300	0	50	78	E2					77
								E3					78
								E4					79
								E5					80

Note: The mass concentration in the table is the percentage of the total mass of solids such as gangue, PFA, and FG to the mass of all paste materials.

## 3 Test results

### 3.1 Trial formulation test results of FG-based paste filling material

**Fig 2:**
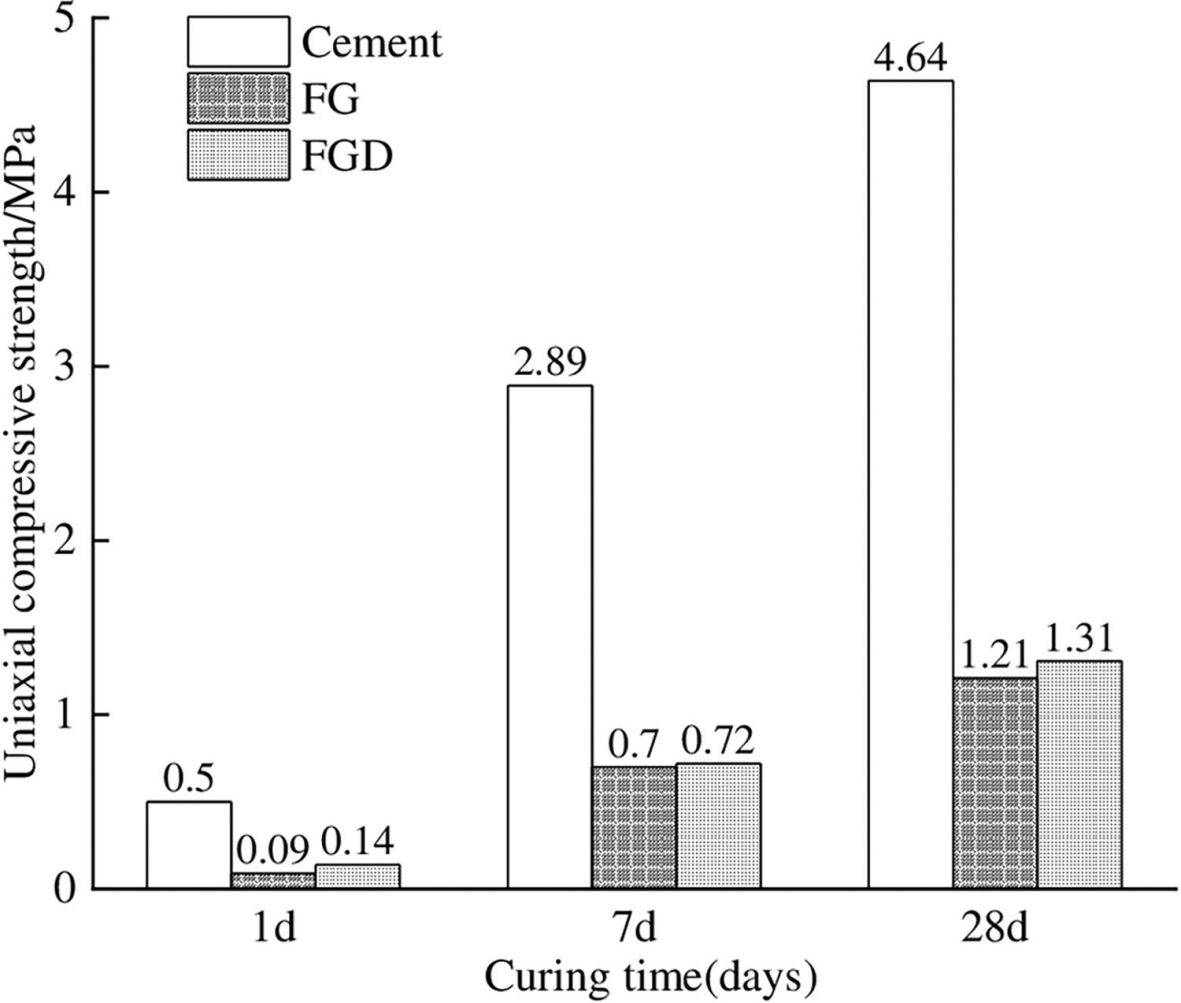
Comparison of the strength of the same proportion when the content of cement, FG, and FGD is 200g.

To show the mechanical properties of materials more intuitively, the tests were carried out using the same proportional substitution of raw materials, as shown in Table 3 for scheme 1~scheme 3 and scheme 4~scheme 6. This contrast test allowed a better comparative analysis of the test results. Fig 2 lists the comparative diagram of the compressive strength of cement, FG, and FGD in the same proportion.

**Fig 3:**
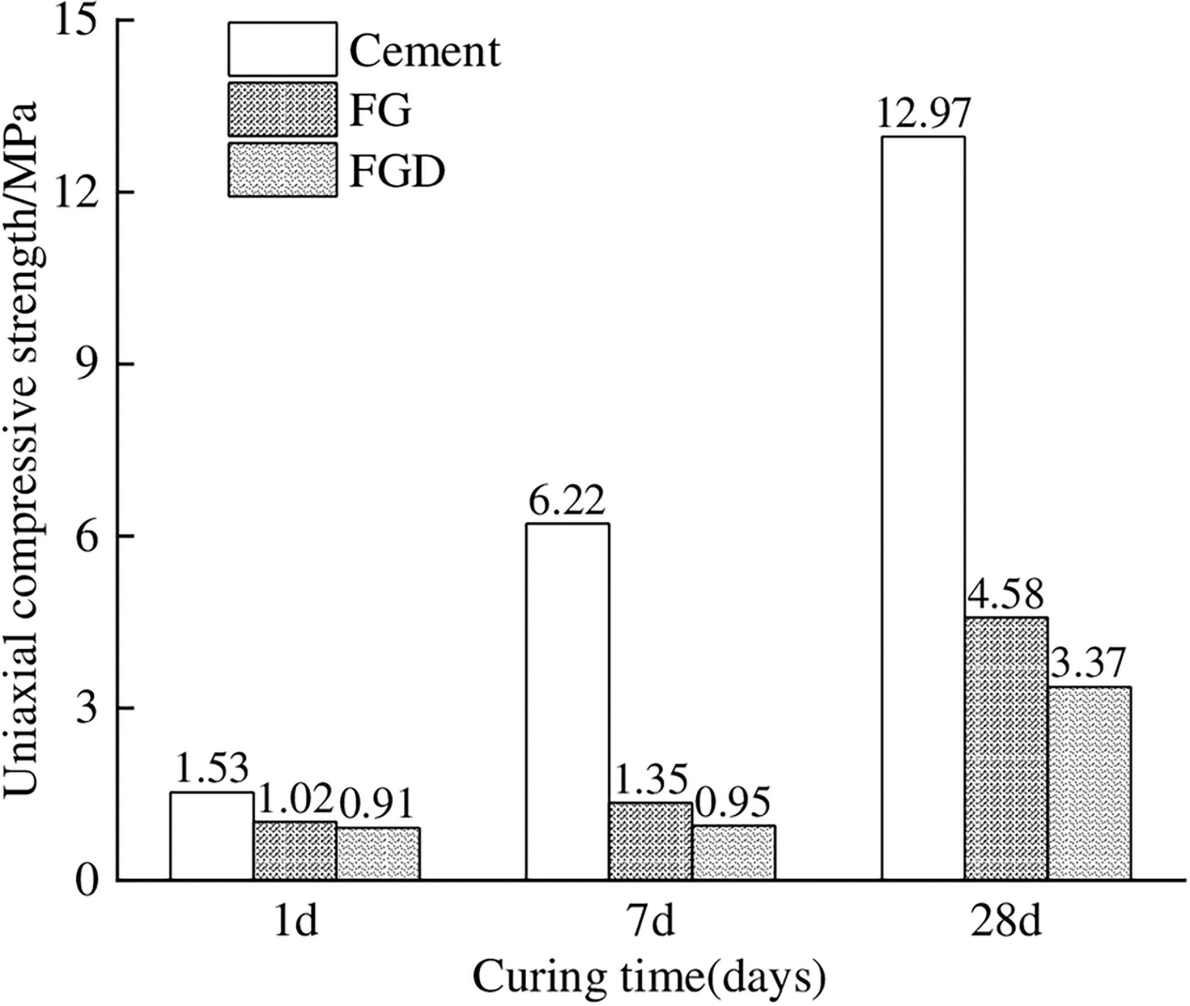
Comparison of the strength of the same proportion when the content of cement, FG, and FGD is 300g.

It can be seen from Fig 2 that the compressive strengths of the same proportion of FG and FGD at all ages of the paste filling material were close to each other, but the ratio was far from that of cement. To achieve the compressive strength of cement in Scheme 1, it was necessary to consider increasing the proportion of cementing materials to enhance the activity of PFA and FG or FGD to increase the hydration reaction rate of the material and enhance the compressive strength of the material at all ages. Accordingly, scheme 4~scheme 6 in Table 3 was developed, and the corresponding results were measured as shown in Fig 3.

**Fig 4:**
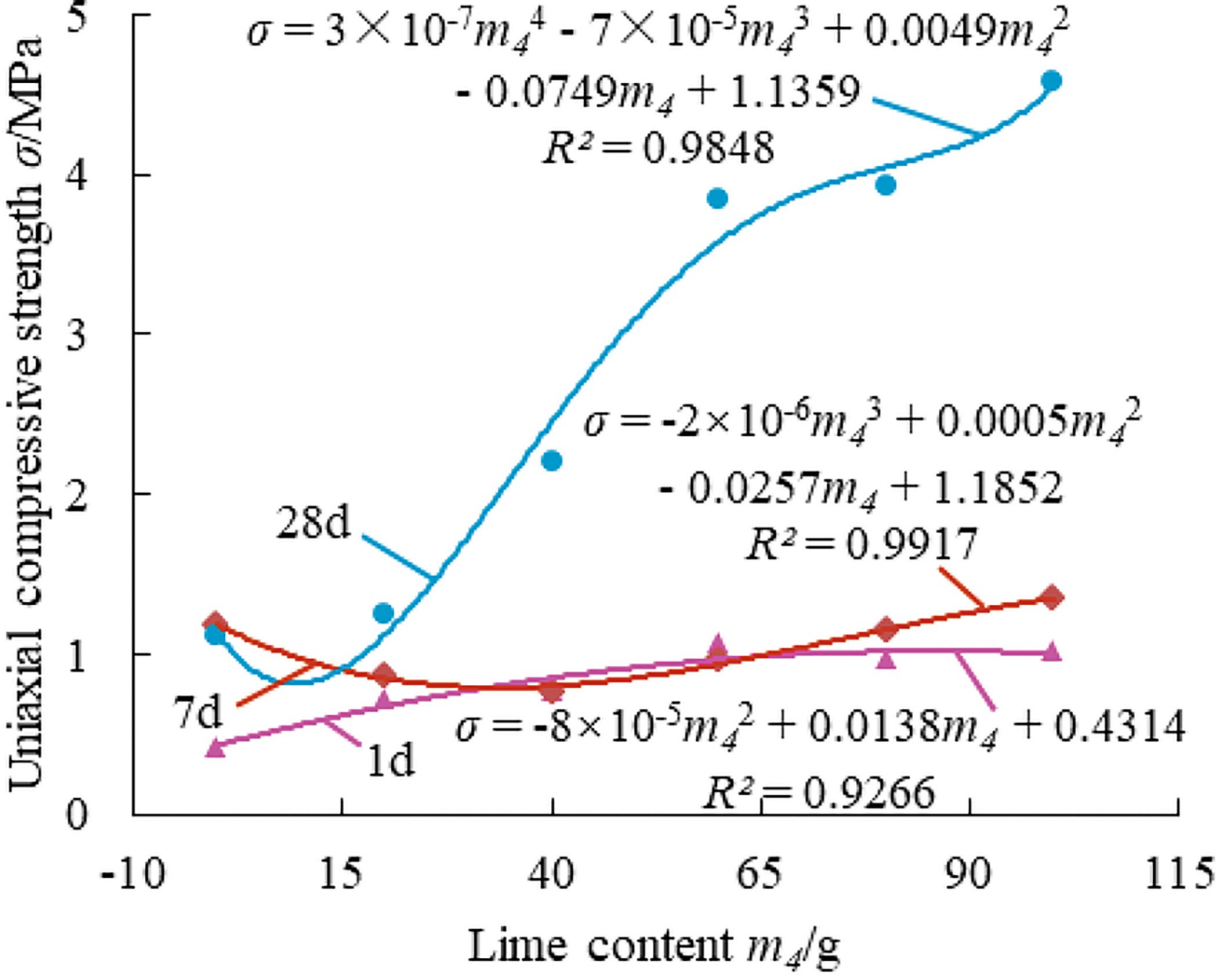
Effect of lime content on the strength of paste material.

**Fig 5:**
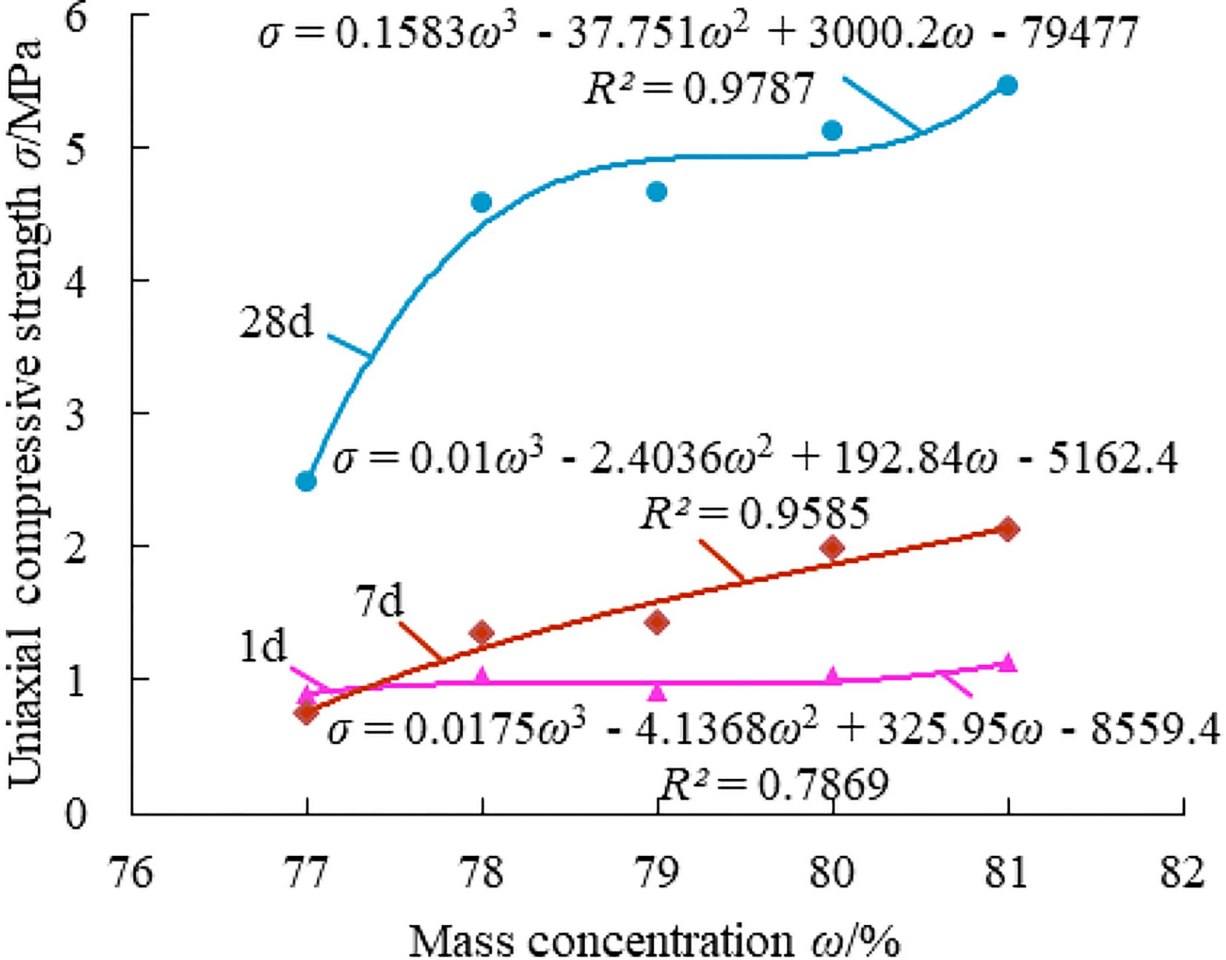
Effect of mass concentration on the strength of paste material.

Compared with Fig 2, it is obvious in Fig 3 that the compressive strength of the material increases significantly at all ages. Although there is still a certain gap between FG and FGD content of 300g and the same proportion strength of cement, it is slightly higher than the compressive strength of cement at all ages in scheme 1, reaching the mechanical properties that can replace cement. By further comparing the rules in Figs 2 and 3, it could be found that the uniaxial compression strength of fluorine gypsum-based filler was less than that of desulfurization gypsum when the content was 200g, but greater than that of desulfurization gypsum when the content was 300g. The reason was that the raw materials of test filling body also contained fly ash, quicklime and coal gangue: the existence of fly ash could enhance the activity of fluorine gypsum and desulfurization gypsum to a certain extent, but the amount of fluorine gypsum consumed was slightly larger than that of desulfurization gypsum. When the content of fluorine gypsum was low, the calcium required for the chemical reaction of filling materials would be insufficient to a certain extent, resulting in the corresponding reduction in the strength of filling body. The hydration reaction of quicklime and fluorine gypsum could enhance the strength of filling body accordingly, and the effect was more obvious with the increase of content. The larger the proportion of coal gangue, the smaller the proportion of cementing material in the same volume test rock sample, which indirectly led to the corresponding reduction of fluorine gypsum content proportion, and also led to the reduction of cementing ability of the filling body. To sum up, although the content of fluorine gypsum and desulfurization gypsum in Fig 3 was greater than that in Fig 2, which could improve the overall strength of the filling body to some extent, the reduction of coal gangue proportion and the large content of quick lime would lead to the enhancement of the effective chemical reaction of fluorine gypsum, and ultimately lead to the uniaxial compressive strength of the fluorine gypsum-based filling being greater than that of desulfurization gypsum-based filling. As a result, while the proportion of fluorine gypsum and desulfurization gypsum in the material in Fig 3 is the same, the performance of fluorine gypsum is superior, and the price of fluorine gypsum is lower and it has higher economic value. Therefore, related tests of desulfurization gypsum will not be carried out in the future. To further optimize the ratio of filling materials, the method of fixing the content of coal gangue, PFA, and FG as the main materials and changing the content of lime and mass concentration is adopted to determine its optimal content. Corresponding data were measured and fitted, and the results were shown in Figs 4 and 5.

Fig 4 shows that the fitting curve of 1 day curing age presents a slight convex curve, indicating that the content of lime contributed to the growth of the compressive strength of the material at 1 day, but the effect on the compressive strength of the material at 1day tends to level off when the lime content reached 60g. The fitted curve at the curing age of 7 days is downward convex, and a turning point occurred when the content of lime reached 40g. When the content of lime is less than or equal to 40g, the compressive strength of the material at 7 days gradually decreases as the content of lime increases; When the content of lime is greater than or equal to 40g, the compressive strength of the material at 7 days gradually increases as the content of lime increases. The fitting curve at the curing age of 28 days is in the shape of an "S". With the increase of the content of lime, the compressive strength of the material at 28 days gradually increases, and the growth tends to be gentle when the content of lime reaches 60g. Although the mechanical properties will be improved when the content of lime is greater than or equal to 60g, the increased range is small and the price of lime is high, which will increase the cost burden of filling materials. Therefore, the content of lime is selected as 50g.

From Fig 5, the strength of the material at all ages increases significantly with the increase of mass concentration, and the fitting curve is relatively flat when the curing age is 1 day, indicating that the change of mass concentration has no effect on the compressive strength of the material in 1 day. The fitting curve at the curing age of 7 days is a monotonically increasing line, indicating that the mass concentration had a significant effect on the compressive strength of the material at 7 days. The material’s fitting curve showed a turning point at 28 days when the mass concentration reaches 78%, which is the moment at which the compressive strength dramatically increases. When taking into account the material’s compressive strength, the mass concentration of the material at 78% produces the best cost performance.

According to the above-mentioned test results, the proportioning of FG-based paste filling materials is 1000g coal gangue, 300g PFA, 300g FG, 50g lime, and mass concentration is 78%.

**Fig 6:**
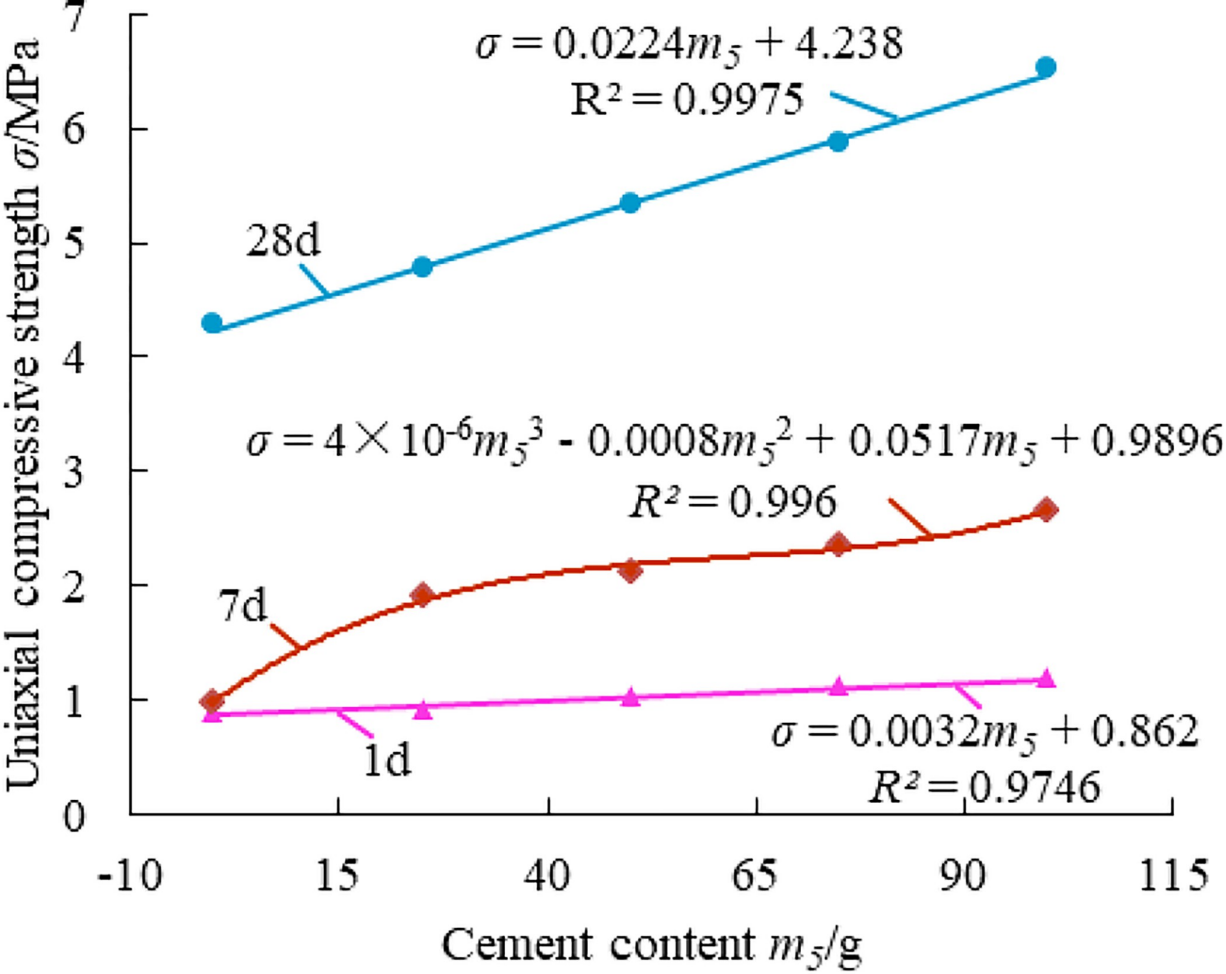
Effect of adding a small amount of cement on the strength of the paste material.

Fig 6 shows that when the curing age is 1 day, the slope of the fitting curve is modest, indicating a small improvement in the material’s compressive strength due to the addition of cement. When the curing age is 7 days, the fitted curve has the shape of an "S" showing that the growth of the material’s compressive strength at this age is minimal when the cement content is less than or equal to 75g, and significant when the cement content is greater than or equal to 75g. The material’s compressive strength improves linearly with an increase in cement content at 28 days, according to the fitted curve, which is linear and has a steep slope at the curing age of 28 days. The reason why the hydration reaction of cement is slow in the early stage and mostly concentrated in the late stage is that ettringite, which adhered to the surface of cement particles, was produced by the reaction of CaSO_4_∙2H_2_O in FG with the hydration product of calcium aluminate in cement. When the cement concentration is 0g, the material’s strength at 28 days can be as high as 4MPa; however, when the cement amount increases, the material’s strength rises by 52.6%, from 4.28MPa to 6.53MPa. It can be seen that by adding a small amount of cement, the applicability of FG-based paste filling materials can be greatly improved to meet their different requirements for the mechanical properties of the materials under different mining geological conditions.

### 3.2 Strength properties of FG-based paste filling material

**Fig 7:**
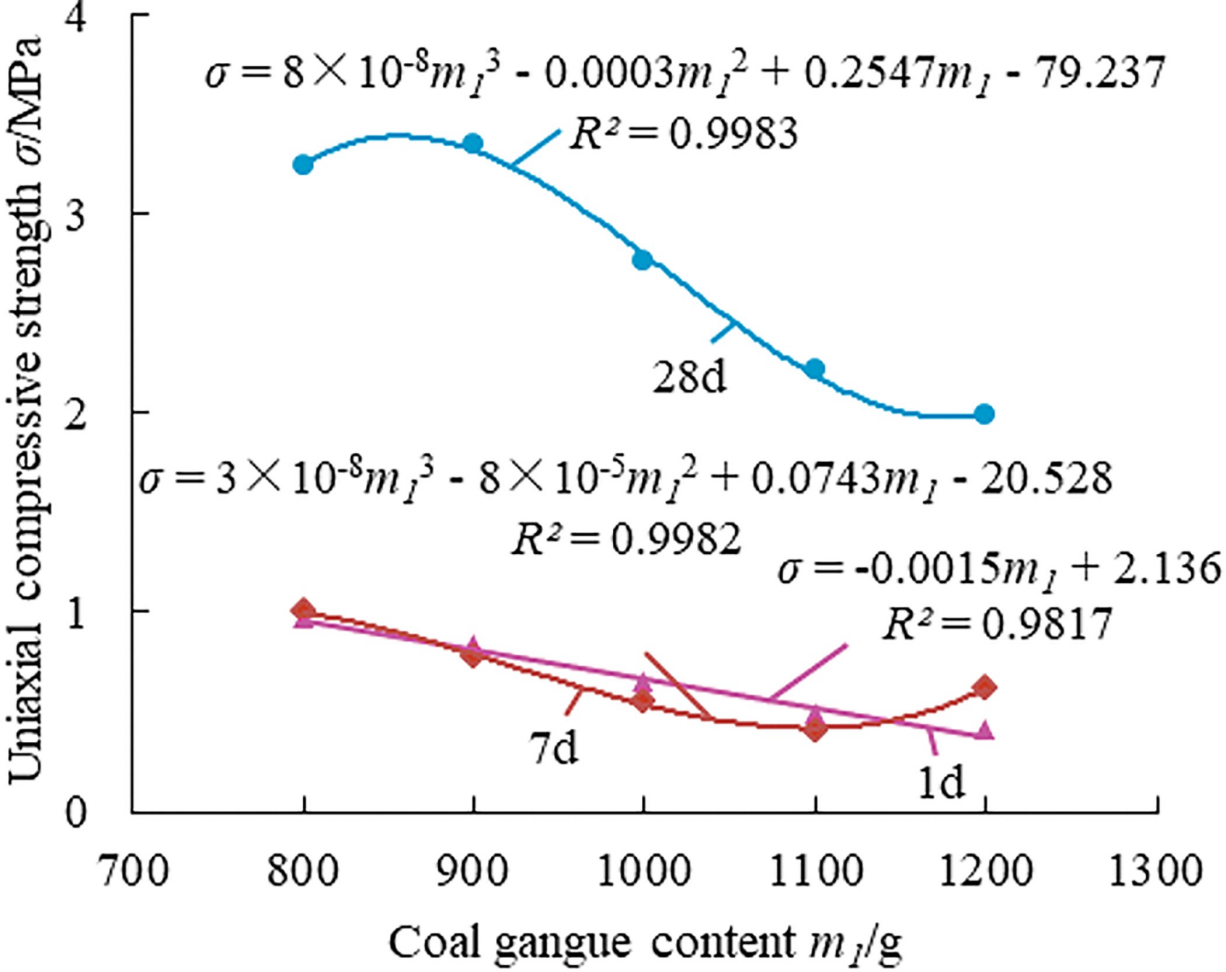
Effect law of gangue content on the strength of paste material.

**Fig 8:**
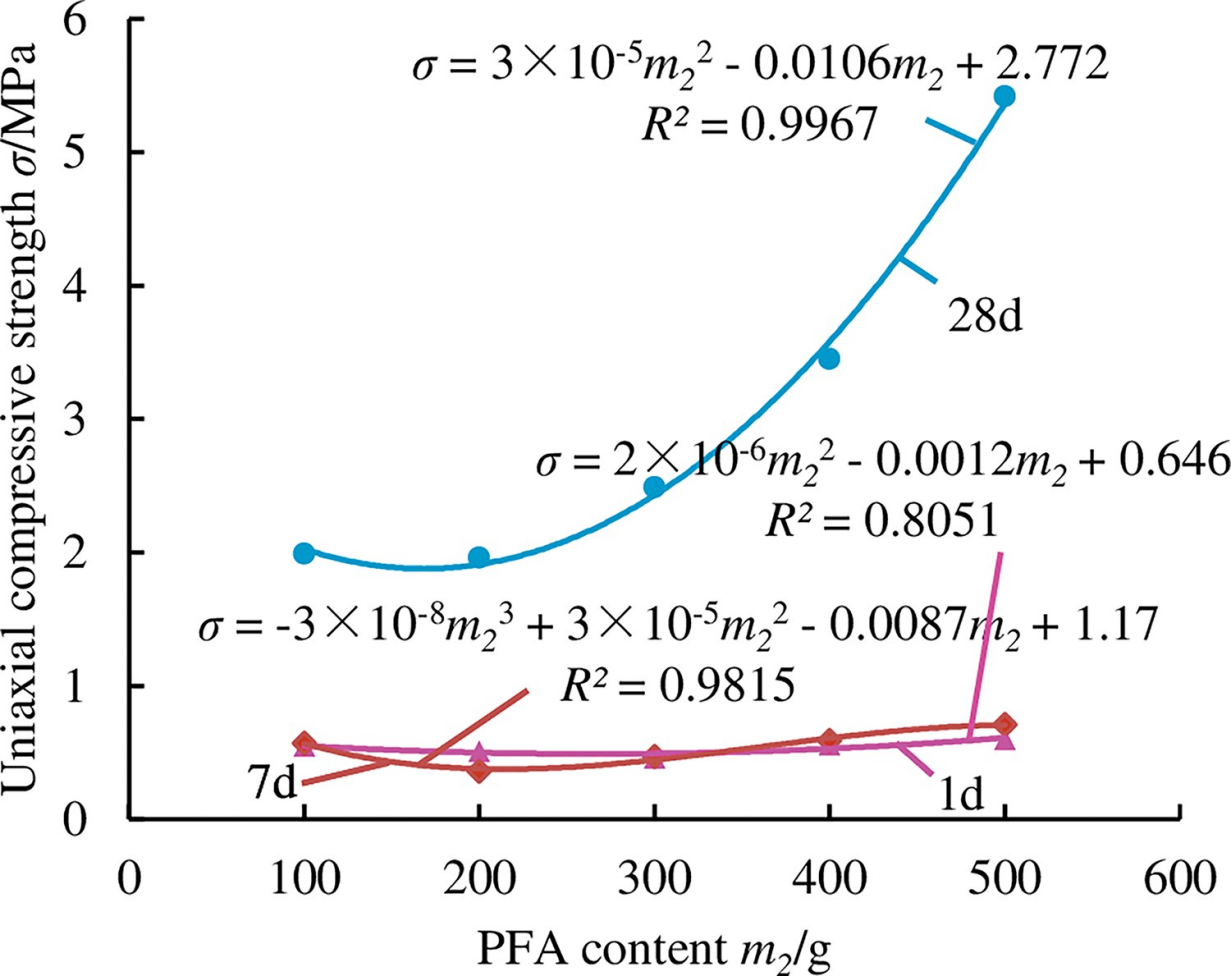
Effect law of PFA on the strength of paste material.

**Fig 9:**
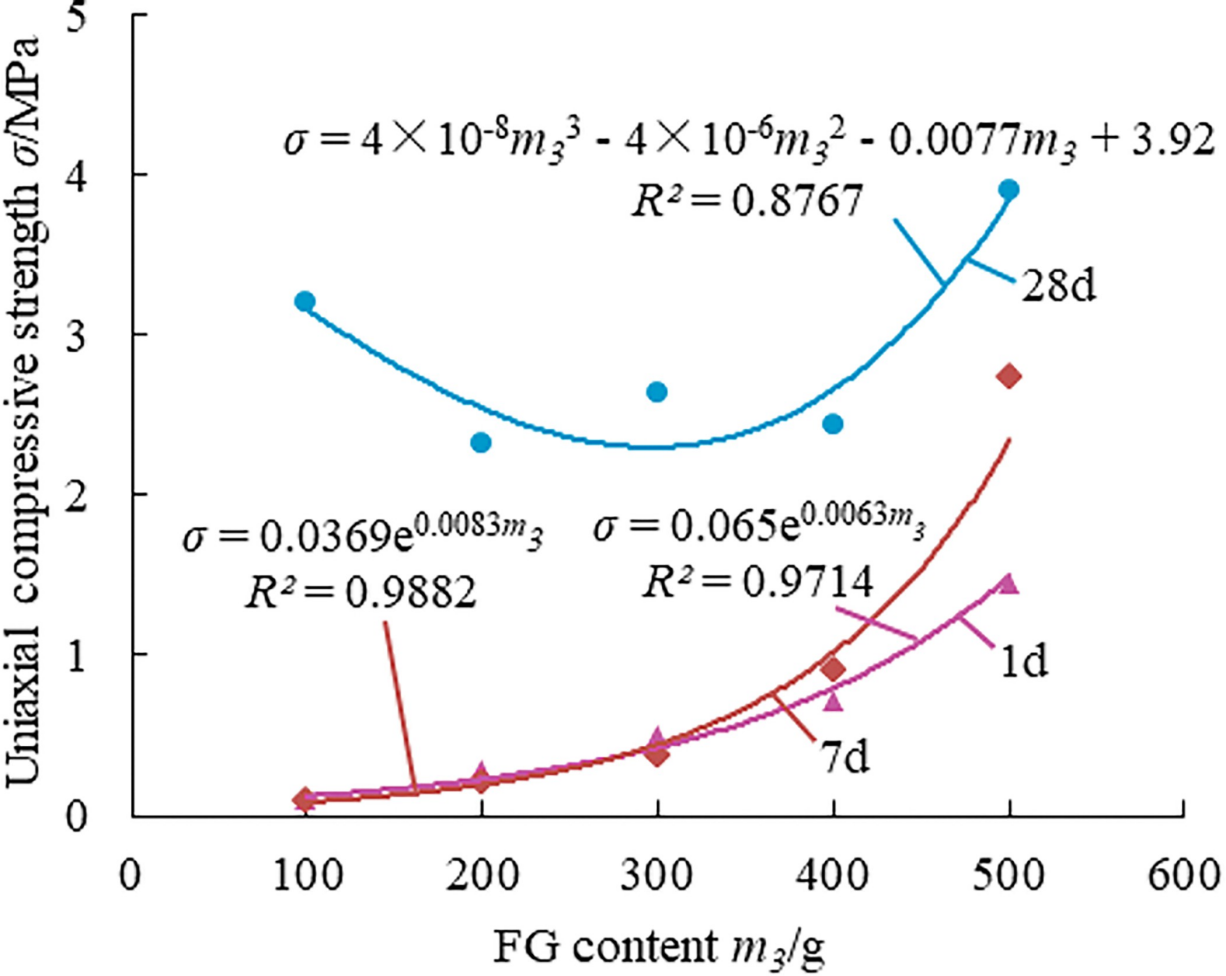
Effect law of FG on the strength of paste material.

**Fig 10:**
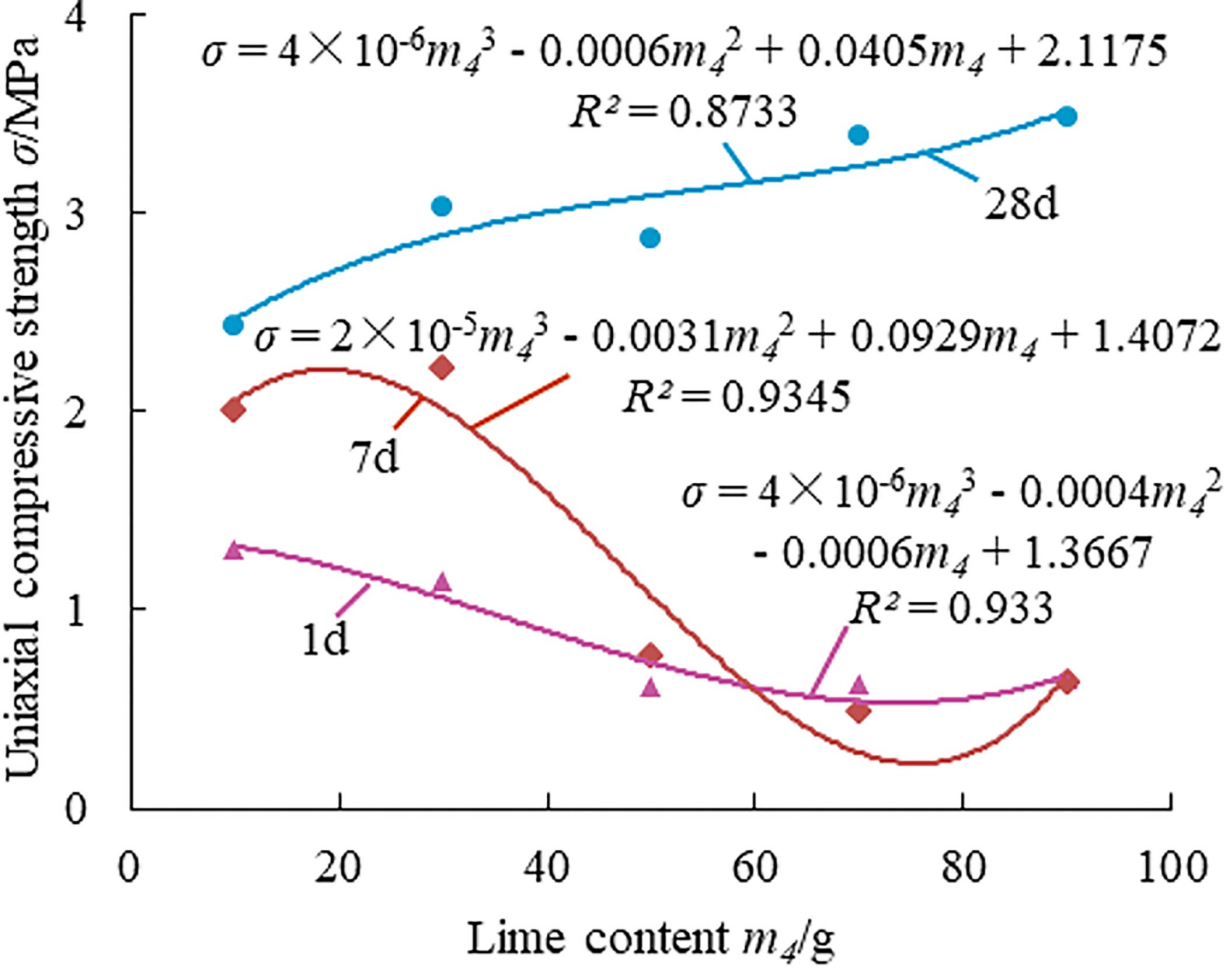
Effect law of lime on the strength of paste material.

**Fig 11:**
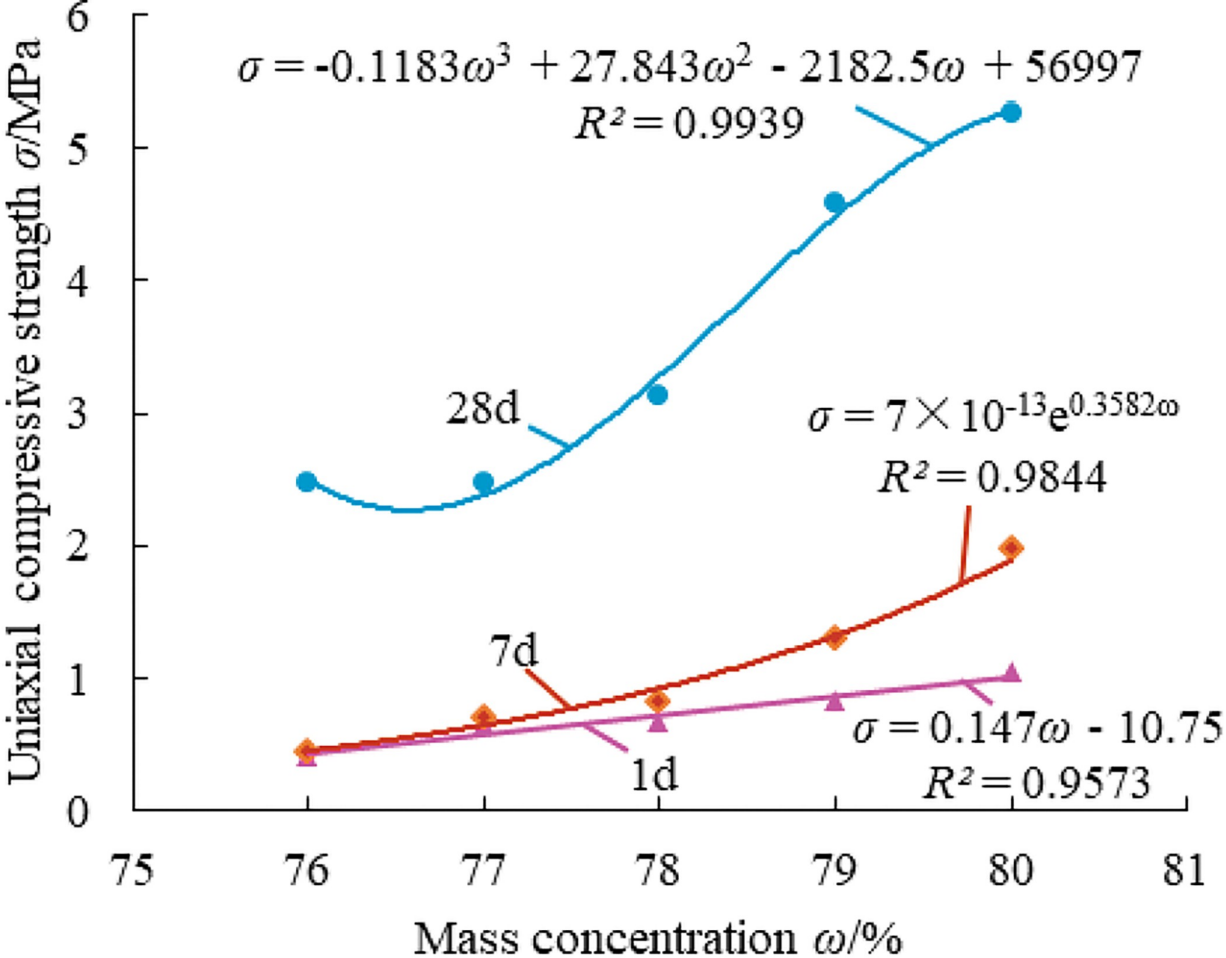
Effect law of mass concentration on the strength of paste material.

Mine paste filling material must meet specific fluidity and stability requirements as well as pre-strength and post-strength requirements in order to achieve rapid filling, achieve the goal of controlling surrounding rock and overlying rock, and play a specific role in supporting the overlying rock in the mining region in time to enable the safe recovery of the mine and prevent and control surface erosion. Investigating how each raw material affects the mechanical properties of the filling material can help to get a better filling result. Figs 7–11 show the influence of each raw material on the compressive strength of the new FG-based paste filling material.

Fig 7 shows that with the increase of coal gangue content, the compressive strength of each age in the system gradually decreases, which indicates that the contribution of coal gangue to the compressive strength of the filler was very small. As the main raw material, the increase of coal gangue content means that the proportion of cementing material decreases. The cementing property becomes inadequate and the material’s overall strength declines when the amount of cementing material is reduced to a particular degree.

Fig 8 shows that the system’s compressive strength at 1 and 7 days was 0.57 MPa on average, with the PFA component having little to no impact. However, it has a significant effect on the strength of the material at 28 days, and when the PFA concentration climbs from 100g to 500g, its compressive strength increases from 1.96 MPa to 5.42 MPa, with an increase of 176.5%. The low strength in the early stage is caused by the low activity of PFA and FG as well as the sluggish reaction rate in the early stage. The increase of strength in the later period is due to the occurrence of volcanic ash reaction and micro-aggregate effect, which is the reaction of SiO_2_ and Al_2_O_3_ in PFA with Ca(OH)_2_ to generate hydrated calcium silicate, hydrated calcium aluminate, etc. Additionally, the PFA is equally dispersed throughout the slurry and the majority of the particles are microbeads, which enhance the slurry’s general homogeneity and subsequently raise the material’s overall compactness, thereby the strength of the material will be significantly improved.

Fig 9 presents that the compressive strength of FG paste material increases gradually as the amount of the material is increased. The compressive strength increases by 1340% and 2944%, respectively, when the weight of the material is increased from 100g to 500g. The main cause of this rise in compressive strength is anhydrite hydration, which results in the formation of dihydrate gypsum. The compressive strength of the paste material at 28 days first declines and subsequently increases as FG content rises. The cause of this occurrence is the volcanic ash reaction that takes place at this stage of PFA. In order to generate calcium alumina, some gypsum must be consumed, which reduces the strength of the material, but this effect is minimal in the presence of sufficient gypsum.

The compressive strength of the paste filling material increases by 10.5% at 30g of lime compared to 10g of lime, as shown in Fig 10, and gradually decreases with increasing lime content at 1 and 7 days. The compressive strength of the filling material at 28 days steadily increases as the lime content rises; as compared to the lime content of 10g, it rises by 24.7% at content 30g and by 43.2% at content 90g. The main cause of the increase in strength is the hydration of lime to create Ca(OH)_2_, which is combined with SiO_2_ and Al_2_O_3_ in PFA and CaSO4 in FG to create C-S-H gel and ettringite.

Fig 11 demonstrates that when mass concentration grows, the strength of paste material at each age significantly rises, with the strength at 28 days rising to the highest level. Insufficient mass concentration of the paste filling material will result in poor strength, water secretion, and segregation; excessive mass concentration will result in the excessive viscosity of the material, which is unfavorable for mixing and conveying. As a result, the mass concentration of material should be raised as much as feasible while still maintaining the flow and transportation properties of the material.

To summarize, as the main raw material, coal gangue didn’t react with other raw materials, and its greater content caused less the proportion of cementing material, resulting in a corresponding reduction in the strength of backfill. The increase of PFA modulated the gelation of the paste material, which to some extent enhanced its cementing properties. The major principle was that the main components SiO_2_ and Al_2_O_3_ in PFA reacted chemically with Ca(OH)_2_ in the raw materials to produce hydrated calcium silicate and hydrated calcium aluminate, as follows:

$$2SiO_{2}+3Ca(OH{)}_{2}\to 3CaO\cdot 2SiO_{2}\cdot 3H_{2}O$$ (1)

$$Al_{2}O_{3}+3Ca(OH{)}_{2}+3H_{2}O\to 3CaO\cdot Al_{2}O_{3}\cdot 6H_{2}O$$ (2)

**Fig 12:**
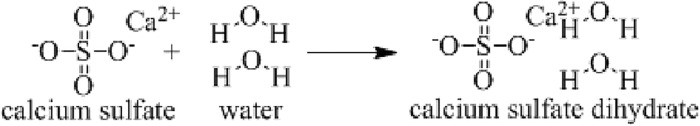
Schematic diagram of the chemical reaction changes of FG crystals.

The hydration reaction of lime and FG also enhanced the overall strength of the paste backfill by changing the chemical composition of the raw materials, such as the formation of C-S-H gel, etc. The schematic diagram of the chemical reaction changes of FG crystals is shown in Fig 12.

The amount of mass concentration characterized the percentage of solid material in the paste raw material. Too large or too small will produce factors that are not conducive to the performance of the paste and should be formulated according to the actual needs. Accordingly, based on the formation mechanism of the new FG-based paste filling material, the filling material suitable for the geological conditions of the mine can be formulated by blending the proportion of each raw material.

### 3.3 Flow characteristics of FG-based paste filling material

**Fig 13:**
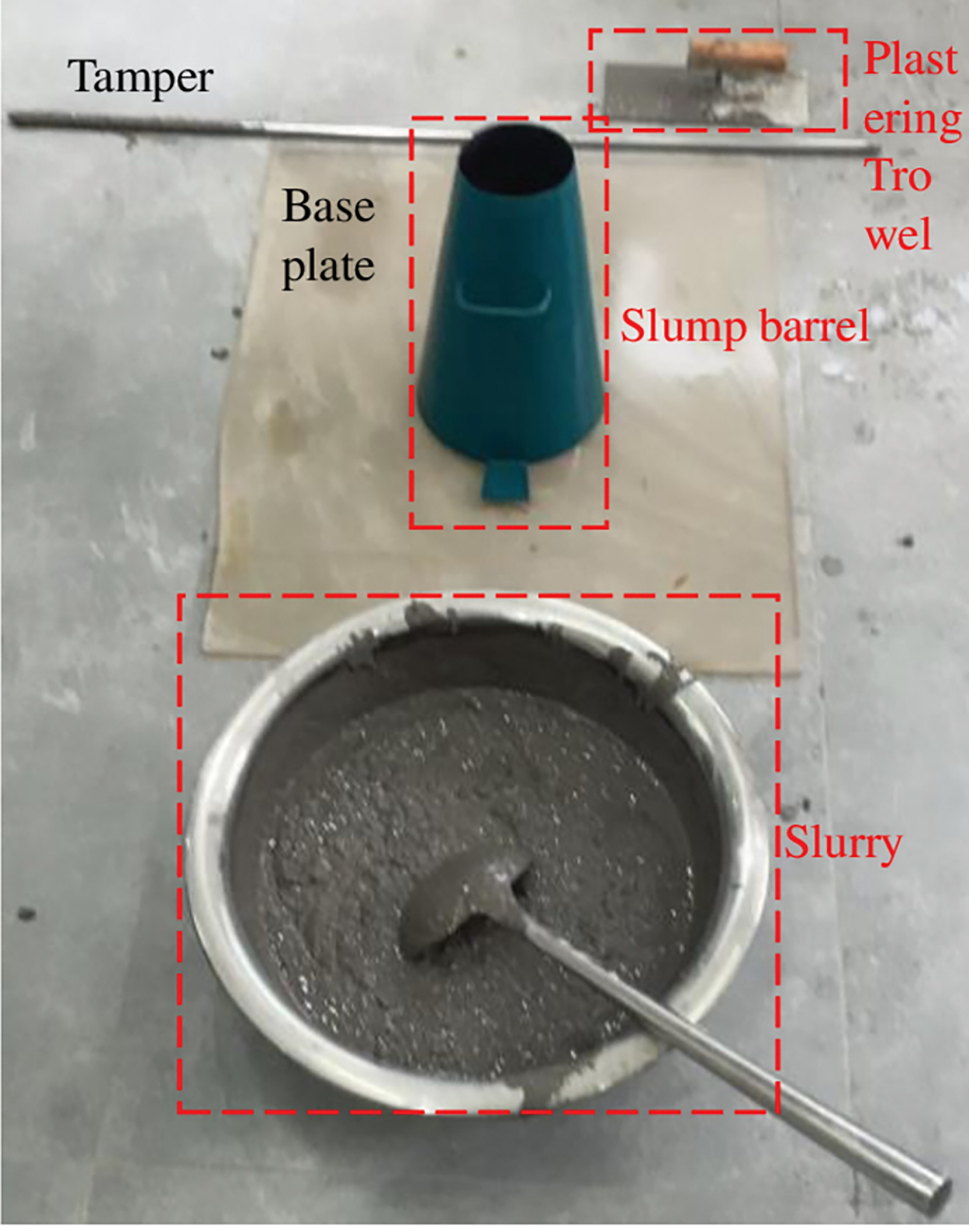
Test equipment for flow performance.

According to GB/T 50080–2016, the slump and expansion of the material were monitored in order to examine the flow characteristics of the FG paste filling material. The test apparatus is displayed in Fig 13.

**Fig 14:**
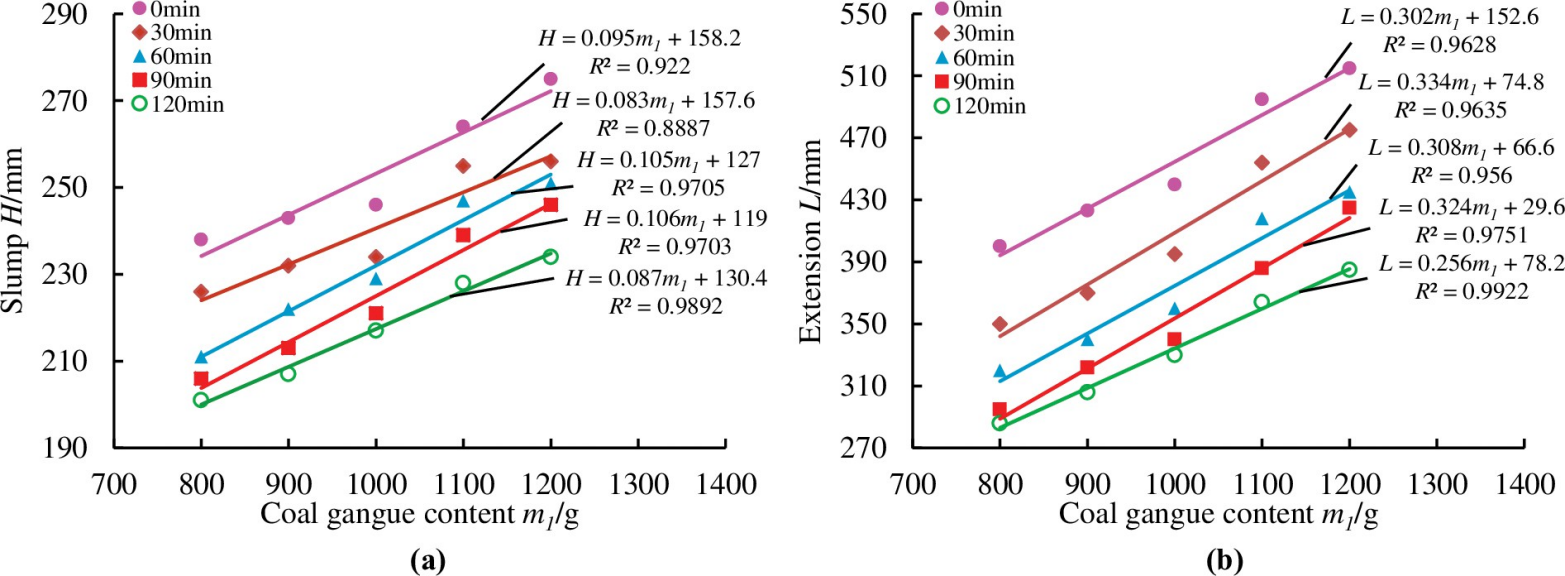
Effect of coal gangue content on material slump and expansion over time.

**Fig 15:**
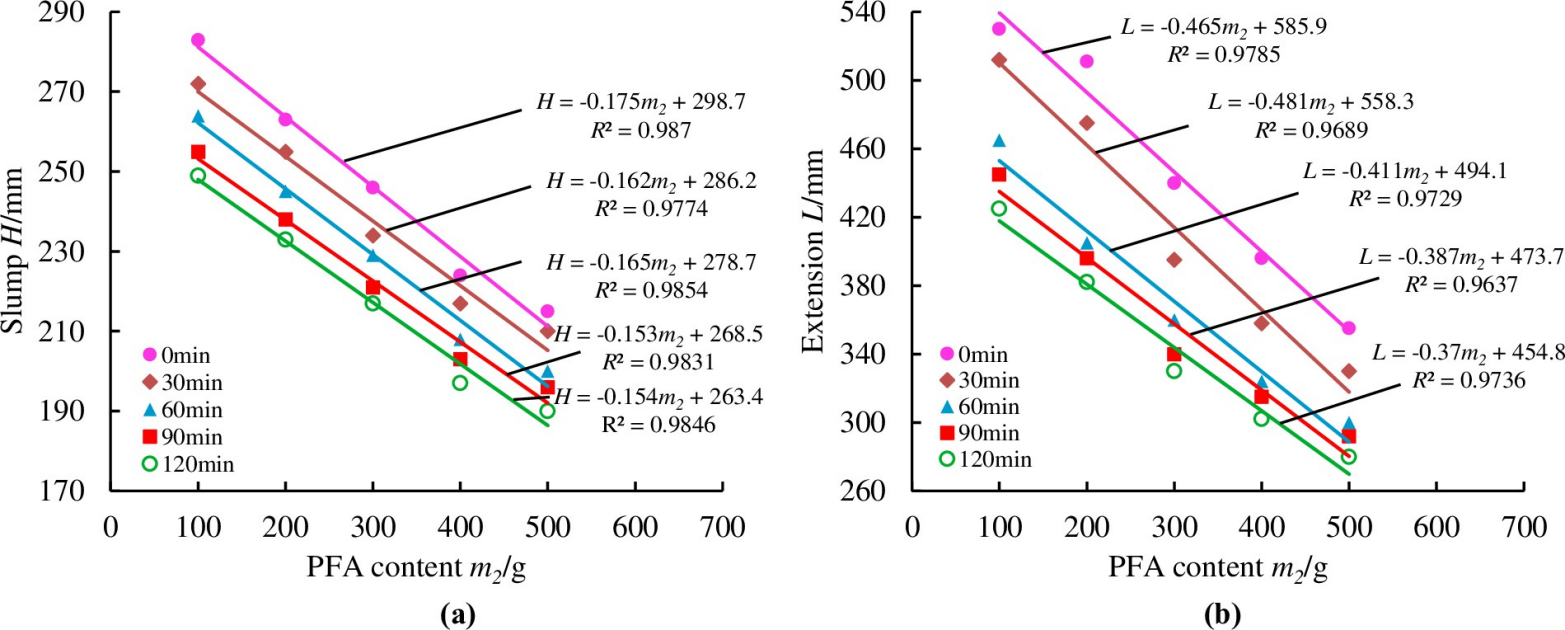
Effect of PFA content on material slump and expansion over time.

**Fig 16:**
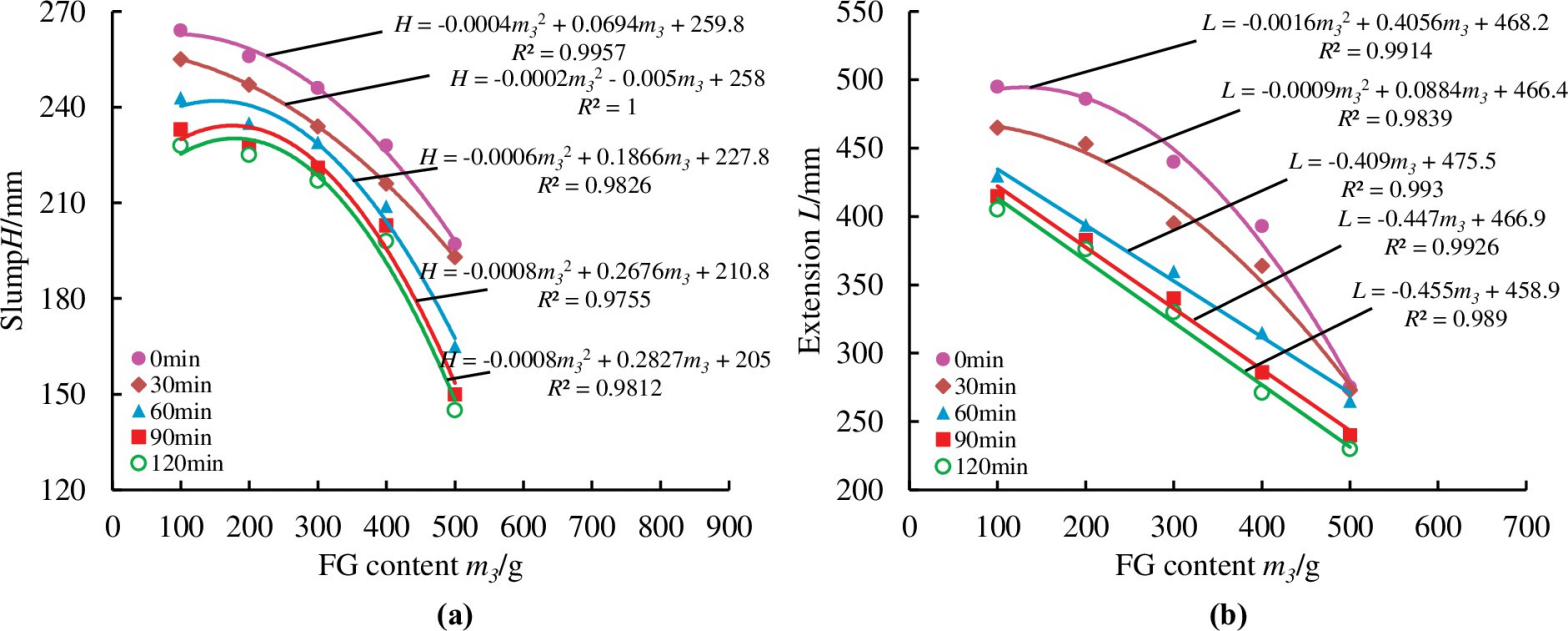
Effect of FG content on material slump and expansion over time.

**Fig 17:**
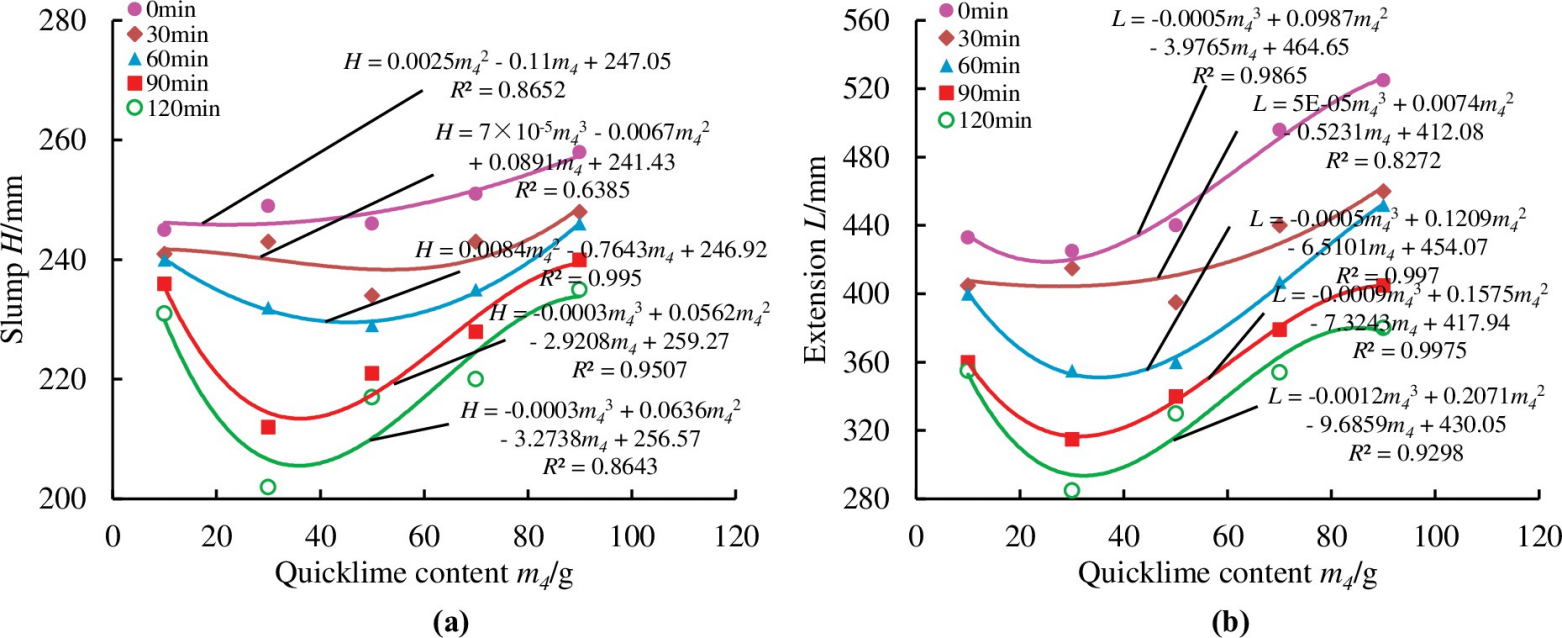
Effect of lime content on material slump and expansion over time.

**Fig 18:**
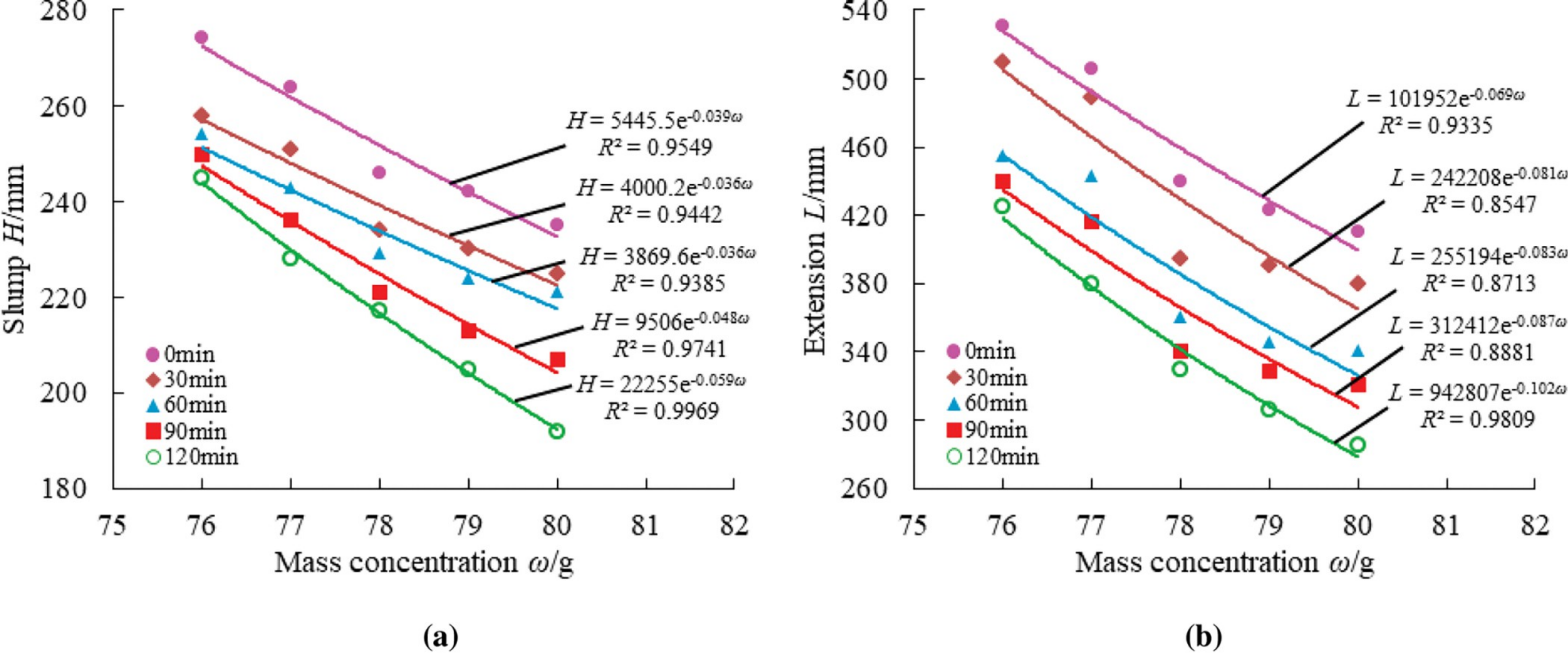
Effect of mass concentration on material slump and expansion over time.

To more precisely reflected the loss of material fluidity over time, measurements were taken at half-hour intervals from the first measurement, for a total of five measurements at 0min, 30min, 60min, 90min, and 120min. As illustrated in Figs 14–18, pertinent data were measured and fitted. The findings indicated that as the testing period was extended, the slump and extension of material diminished.

Fig 14 shows that as the gangue content increases, so do the slump and expansion of the material. In the gangue content between 1000g-1100g, the slump, and expansion change largely because the main raw material ratio is too great, resulting in a fine aggregate ratio that is too small and the cementation performance of the material is insufficient, and the material is prone to segregation and water secretion.

Fig 15 presents that with a PFA content of 100g, the initial slump value of the material can reach 285mm, the greatest value of all the ratios. However, the initial extension value of the material is 530mm, which is equal to a 76% mass concentration at 0 minutes. This shows that the slump and expansion of the material are too great when the PFA content is 100g, causing segregation phenomena, substantial water secretion, and a large amount of coal gangue deposition. Due to the micro-aggregate action of PFA, the slump and expansion of the material are reduced as the PFA concentration rises, which improves the material’s compactness to some extent.

According to Fig 16, the FG content has a smaller impact on the slump and expansion of the material at 100g to 200g, less of an impact at 200g to 300g on the slump, and more of an impact at 300g to 500g on both slump and expansion. Because FG can only serve as a fine aggregate when its content is low, the slump and expansion degree drop is minimal at this time. The activity of PFA will be stimulated and the hydration reaction process will be accelerated when the FG content reaches a particular level, which will result in a significant reduction in the tendency of the material to slump and expansion.

Fig 17 demonstrates that the slump and extension of the materials are not greatly affected by the lime content. The law of influence is not immediately apparent when lime content is between 10g and 50g. When the lime content is between 50g and 90g, the slump and extension of the materials show an upward tendency as the lime content rises. This is because adding too much lime prevents or even inhibits the early hydration process of the material, which causes a slump and expansion phenomenon to be on the rise.

Fig 18 illustrates how the slump and expansion of the material reduce at different rates as the mass concentration rises. The rate of decline is highest between 77 and 78%, while the change in slump and expansion is smaller between 78 and 80%. This might be the case since the material’s hydration reaction rate peaks at 78% mass concentration. Too big or too small is not good for the hydration reaction of the material.

In conclusion, it was clear that each material in the test will have a different degree of influence on the slump and expansion of the material. However, in general, the flowability of the material will be improved to a greater extent with the higher concentration of coal gangue and lime. The flow characteristics of the material are more unfavorable the higher the content and bulk concentration of PFA and FG. Among these, the method by which coal gangue content affected the slump and expansion of material was via altering the ratio of the main raw material to fine aggregate; the major ways that PFA worked were through the effects of micro-aggregate and hydration reactions with other cementing substances. The major function of FG was to act as a fine aggregate and to stimulate the activity of the hydration reaction of PFA; lime controlled the alkalinity acidity of the slurry, which affected the rate of the hydration reaction of material; the mechanism of mass concentration was to alter the rate of the hydration reaction of material by managing the water content, resulting in the development of hydration products like ettringite and C-S-H gels.

## 4 Material macro and microanalysis

### 4.1 Uniaxial compression deformation characteristics

**Fig 19:**
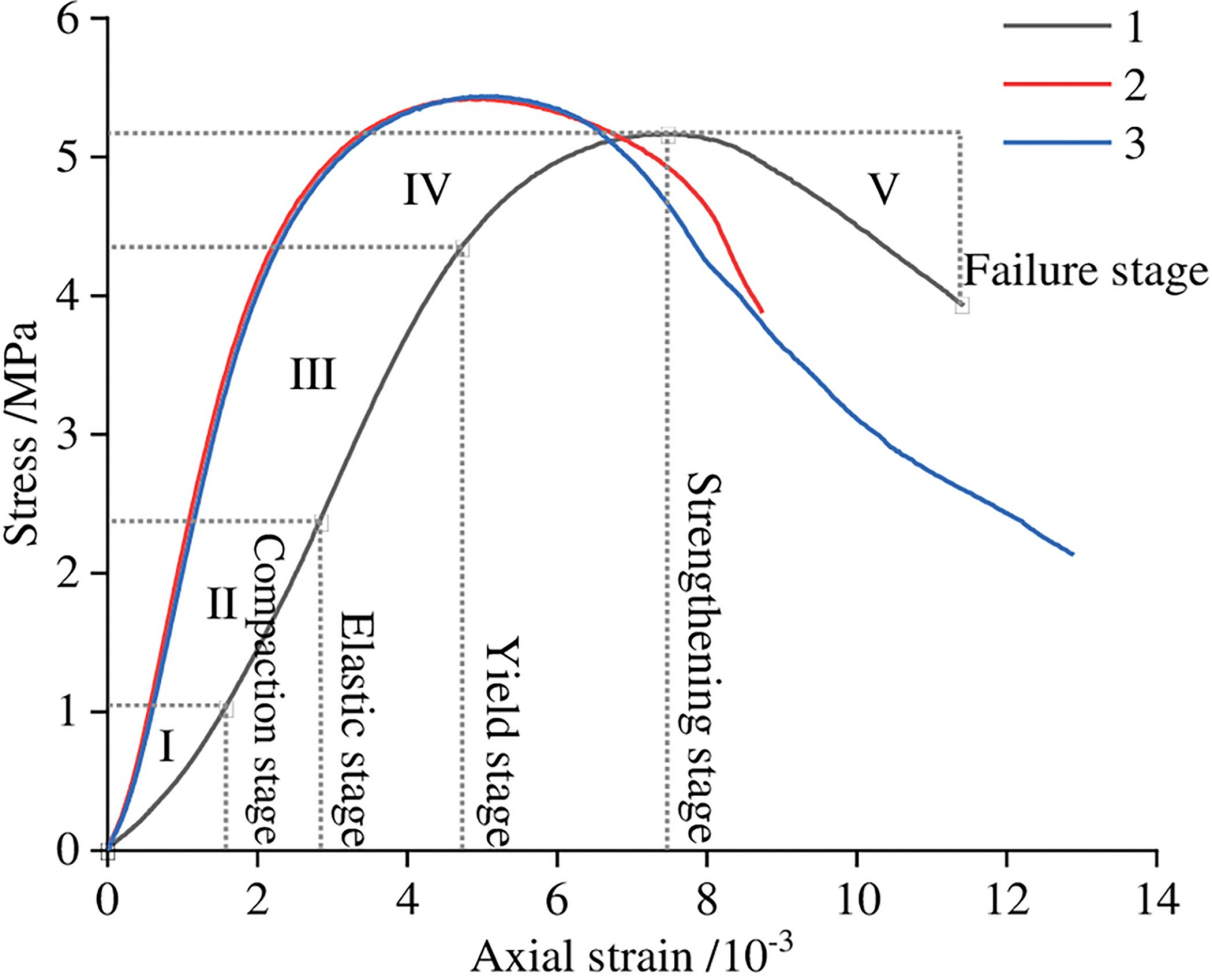
Uniaxial compressive stress-strain curve of the sample at 28 days.

To analyze the uniaxial compression deformation characteristics of the paste filling, a set of typical ratios were selected, namely, 1000g coal gangue, 300g PFA, 300g FG, 50g lime, and mass concentration is 78%, and processed as *ϕ*50mm×100mm cylindrical specimens. Three parallel specimens were set up for the test to reduce the test error. After the samples were kept for 28 days, the uniaxial compression and traditional triaxial compression tests were performed in the RMT-150B electro-hydraulic servo rock mechanics test system. Fig 19 displays the observed data along with the samples’ associated uniaxial compression stress-strain curves for 28 days. It can be seen from the figure that the stress peak value of the sample is 5.17 MPa, 5.42 MPa, and 5.44 MPa, with an average value of 5.34 MPa. According to the characteristics of the stress-strain curve, it can be divided into five stages: compaction stage, elastic stage, yield stage, strengthening stage, and failure stage.

**Fig 20:**
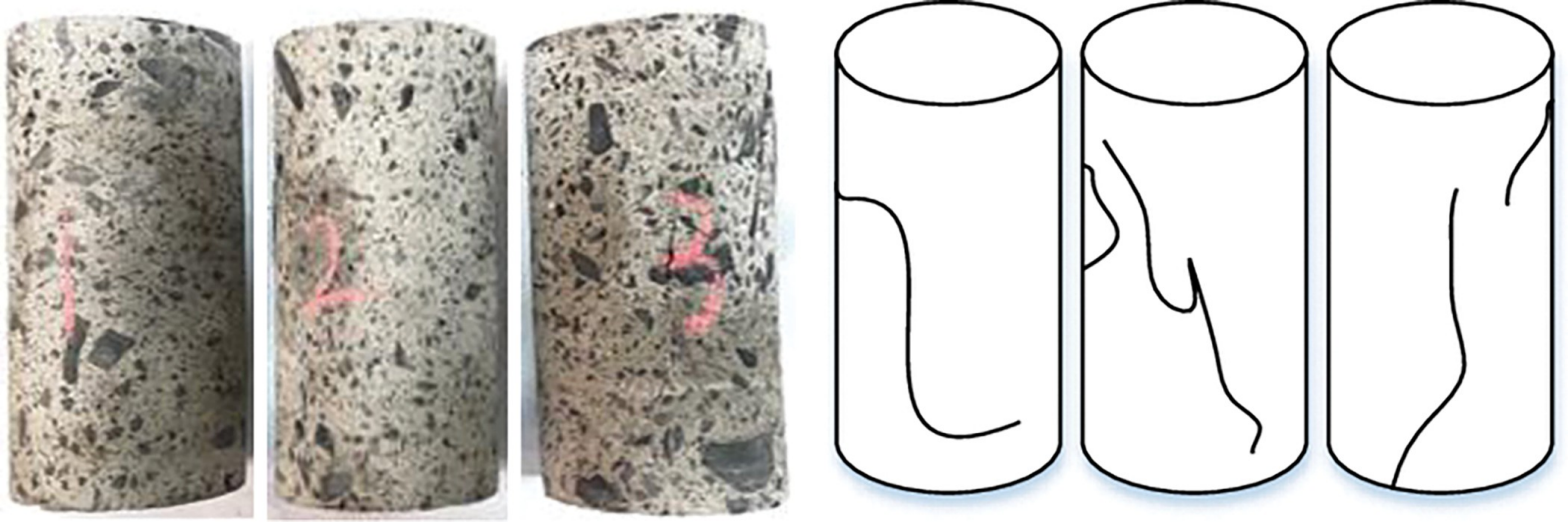
Uniaxial failure characteristics of the sample.

Fig 20 depicts the sample’s compression failure characteristics. The sample’s surface cracks are less noticeable due to the comparatively tiny gangue particle size, improved interaction of gangue and cementing material, and stable internal structure. However, a small portion of the samples’ expansion crack will only occur locally as a result of the gangue’s huge size. The damaged samples’ general structural integrity, which showed only a few cracks, suggested that the developed filling material has strong long-term load resistance.

### 4.2 Triaxial compression deformation characteristics

**Fig 21:**
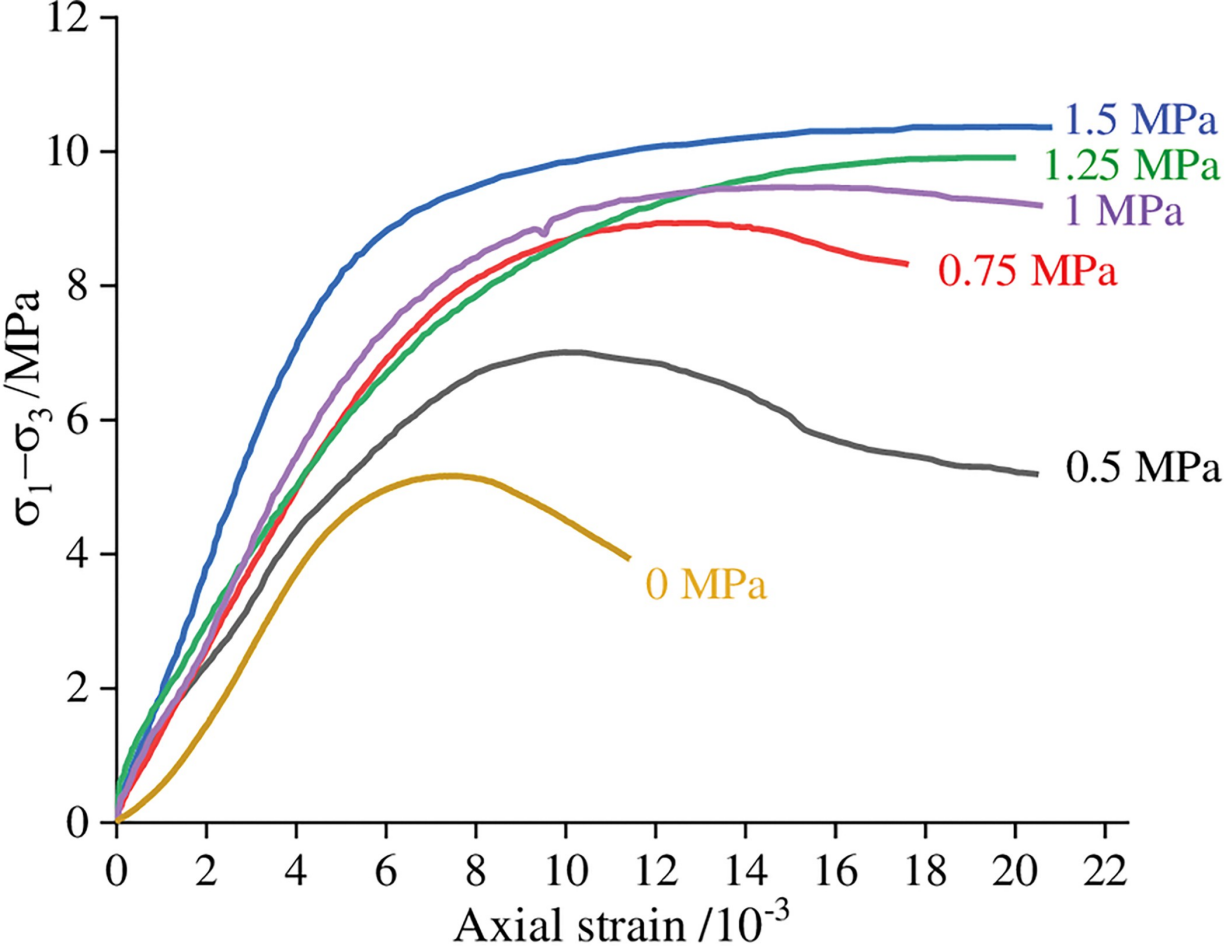
Triaxial compression stress-strain curve of the sample at 28 days.

A triaxial test can more accurately reflect the stress state of the filling material during underground filling since mine filling materials are typically in a three-dimensional stress condition in the mining area. After 28 days of curing, the sample was chosen for a traditional triaxial test, with confining pressures of 0, 0.5, 0.75, 1, 1.25, and 1.5 MPa, respectively. Fig 21 displays the examination outcomes. The figure shows that the post-peak curve of samples gradually becomes softer with increasing confining pressure, indicating that as confining pressure rises, the sample’s post-peak force rises as well. The peak point on the stress-strain curve disappears when the confining pressure hits 1.25 MPa, and the change from plasticity to brittleness exhibits creep features.

### 4.3 Microstructural characteristics

**Fig 22:**
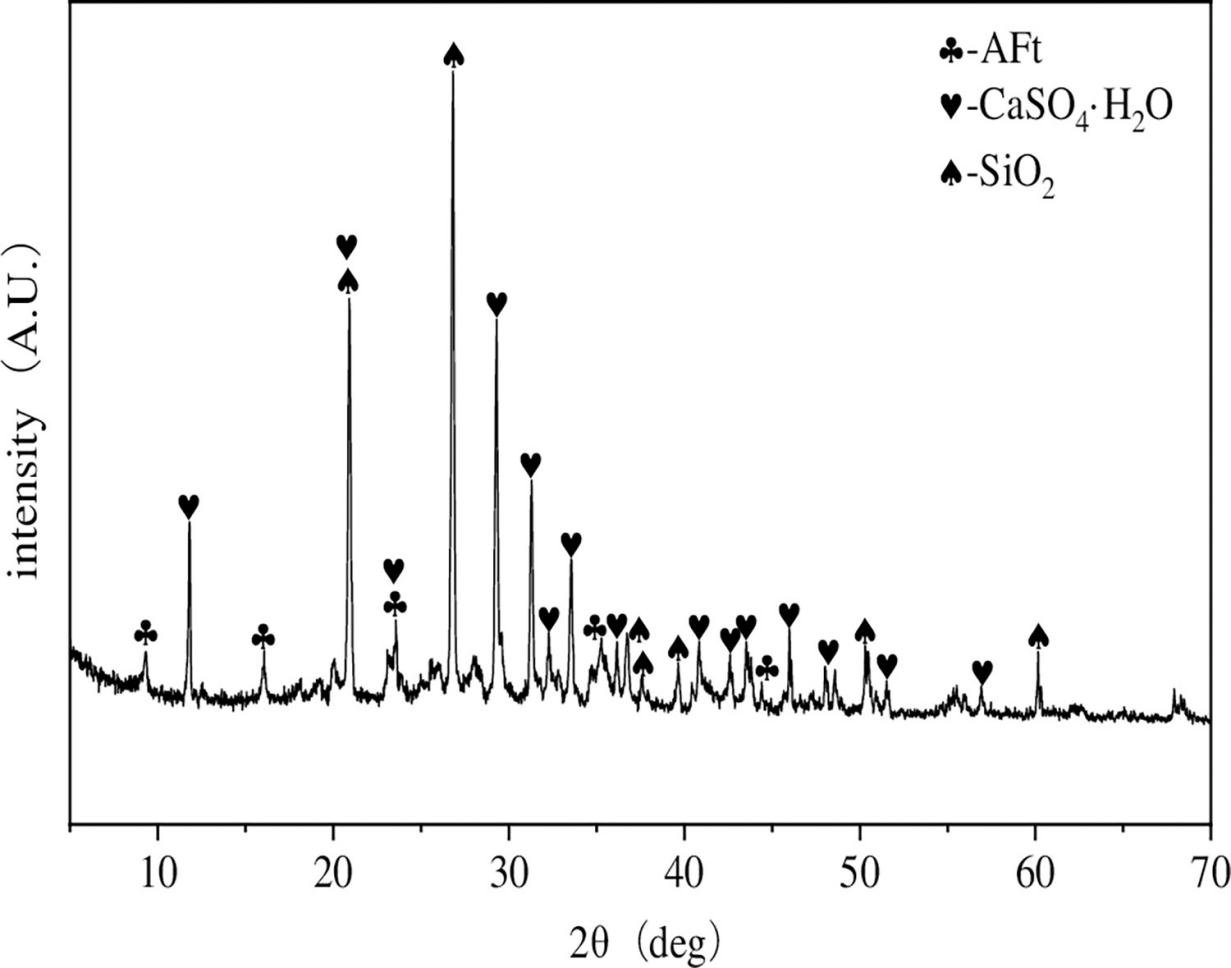
XRD patterns of the materials at 28 days.

**Fig 23:**
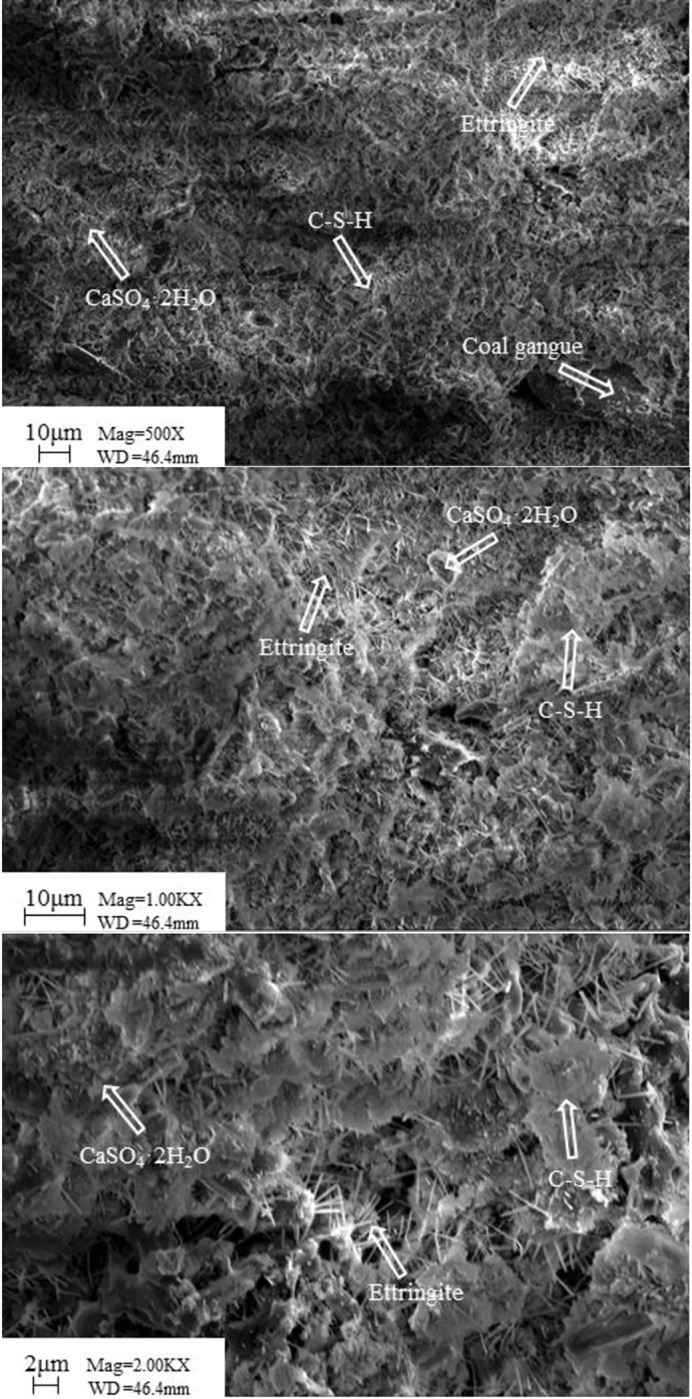
Microstructure of samples at different magnifications for 28d.

Figs 22 and 23 illustrate the results of preparing samples using common ratios in order to explain the hydration products and morphology of hydration products of FG-based paste filling materials from a microscopic perspective.

As shown in Fig 22, ettringite (AFt), calcium sulfate dihydrate (CaSO_4_·2H_2_O), and SiO2 were detected by XRD, indicating that the main hydration products of the paste material were ettringite (AFt), calcium sulfate dihydrate (CaSO_4_·2H_2_O).

The hydration products of FG-based paste filling material, as shown in Fig 23, comprise a significant amount of acicular AFt, C-S-H gel, and certain calcium sulfate dihydrate in some places. Additionally, the material has a compact structure with firmly bound hydration products, giving it significant compressive strength. XRD detected the presence of unreacted SiO2 in the paste. This suggests that there is still room for improvement in the paste’s hardened body strength, which has to be tested over a longer period.

## 5 Discussion

The cement and FGD in the mine paste filling were replaced with the by-product FG as a substitute raw material. Although the strength of the finally developed FG-based paste filling material is lower than that of the cement-based filling material, its compressive strength can eventually reach 4~5 MPa, which can meet the filling requirements of mine goaf, abandoned roadways, etc. At the same time, the main raw material of the developed new paste filling material is the waste coal gangue produced in coal mining, and the cementing raw material is mainly industrial by-product waste FG. The synergistic combination of the two effectively solves the problem of the difficult disposal of waste from related enterprises. Moreover, the developed filling material is filled into the goaf generated during coal mine production, which not only solves the drawback of lacking filling raw materials in the mine, but also effectively improves the stability of the surrounding rocks of the roadway and goaf, and also effectively reduces the surface subsidence caused by mining. Based on the comprehensive consideration of many factors, it can be concluded that the development of a new paste filling material based on the industrial by-product FG has important practical engineering significance. Combined with the physical and mechanical properties of the developed new paste filling material based on fluorine gypsum, coal mine can achieve the purpose of mined-out filling by adjusting the proportion of raw materials such as fluorine gypsum, quicklime and fly ash according to the requirements of filling materials characteristics. Furthermore, the mine can take the lead in measuring the surrounding rock pressure in the gob area, and combine the actual engineering environment and hydrological environment to formulate the physical and mechanical characteristics of filling materials. Then, according to the influence of various raw materials on the strength, fluidity and deformation characteristics of fluorine gypsum-based paste filling materials, the physical and mechanical properties of materials can be improved by adjusting the proportion of various raw materials. The paste filler which can support the surrounding rock in coal mine goaf is prepared.

## 6 Conclusions

A new paste filling material was created using the concept of reusing FG, a byproduct of the preparation of hydrofluoric acid. It can solve the problem of material shortage in mined-out area and abandoned roadway. The following results were reached after doing a compounding ratio test, a single-factor analysis, and macro and micro analyses of the data:

(1) The innovative FG-based paste filling material ratio was established as 1000g coal gangue, 300g PFA, 300g FG, 50g lime, and 78% of mass concentration. Its uniaxial compressive strength at 28 days is approximately 4-5MPa.
(2) Too much coal gangue as the main raw material will weaken the strength of the material; nevertheless, PFA and FG contents considerably increased the material’s strength in the late and early stages, respectively. The lime component decreased the material’s early strength while increasing its late strength; adding a small amount of cement can improve the strength of the filling material; mass concentration has greatly increased the material’s strength at all ages.
(3) The flowability of the material was improved by the increased content of coal gangue and lime, whereas the flowability was negatively impacted by the higher content and mass concentration of PFA and FG.
(4) Under uniaxial compression, the FG-based paste backfill presented five typical stages: compression-density stage, elastic stage, yield stage, strengthening stage, and failure stage, with typical elastic-plastic characteristics. In contrast, the triaxial compression condition showed typical creep characteristics.
(5) By using XRD and SEM techniques, the developed materials were microscopically characterized. The materials’ hydration products included ettringite (AFt), calcium sulfate dihydrate (CaSO_4_·2H_2_O), and hydrated calcium silicate gel (C-S-H).
